# Supplementary material for: The Low-Copy-Number Satellite DNAs of the Model Beetle Tribolium castaneum
Source: Genes (Basel). 2023 Apr 28;14(5):999. doi: 10.3390/genes14050999 (PMC10218199; doi:10.3390/genes14050999)
Supplement: Supplementary file 1 [file genes-14-00999-s001.zip › Supplementary materials/Figure S1.pdf]

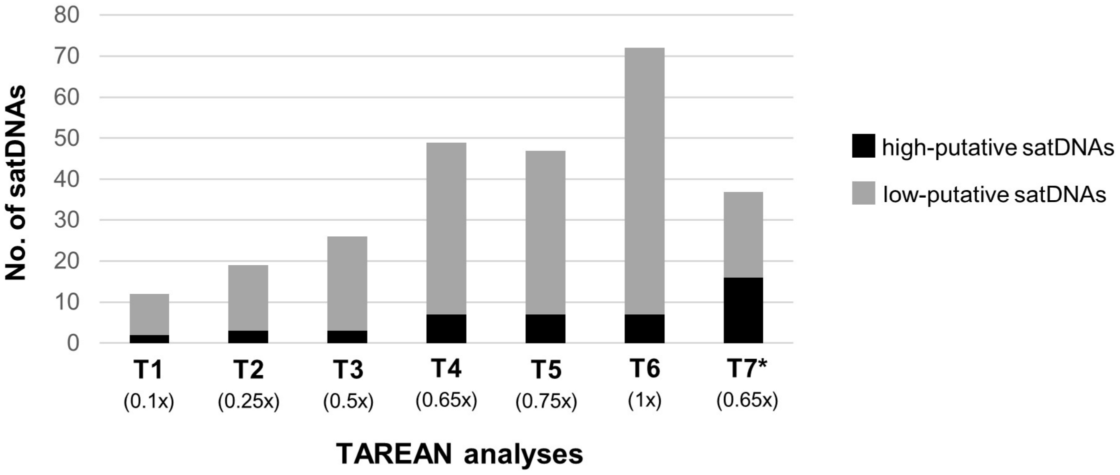

Figure S1. The number of high-putative (black) and low-putative (gray) satellite DNAs identified in the seven TAREAN clustering analyses (T1-T7). Genome coverage, corresponding to the total length of reads used in each TAREAN analysis, is indicated in parentheses. An asterisk marks an analysis in which reads belonging to the major satDNA TCAST were excluded from the initial subset of 1,500,000 reads.
