# Supplementary material for: The Low-Copy-Number Satellite DNAs of the Model Beetle Tribolium castaneum
Source: Genes (Basel). 2023 Apr 28;14(5):999. doi: 10.3390/genes14050999 (PMC10218199; doi:10.3390/genes14050999)
Supplement: Supplementary file 1 [file genes-14-00999-s001.zip › Supplementary materials/Figure S2.pdf]

A)

TCsat14

SatDNA	From	To	Repbase	From	To	Dir	Sim	Pos/Mm:Ts	Score
TCsat14	168	227	HAT-14_Mad	1525	1587	c	0.7903	1.2857	242

```
 168  ATTTCCACAAAAAA--AGTGTG-GTTCAGTTCTAAATATTTTACAAT--AATTTAACTTTTTTAG  227
      ||||||||:||||:--||||||-|||:||--||||||||||:|||||--|| |:|||:||: |||
1587  ATTTCCACGAAAAGCCAGTGTGTGTTTAG--CTAAATATTTCACAATGGAAATCAACCTTCGTAG  1525
```

TCsat15

SatDNA	From	To	Repbase	From	To	Dir	Sim	Pos/Mm:Ts	Score
TCsat15	495	522	Academ-3_LMi	72	99	c	0.9643	1.0000	244

```
495  TCGGAAAGAGCACATCAAAATCTATAAG  522
     |||:||||||||||||||||||||||||
 99  TCGAAAAGAGCACATCAAAATCTATAAG  72
```

SatDNA	From	To	Repbase	From	To	Dir	Sim	Pos/Mm:Ts	Score
TCsat15	663	693	Transib-N1_CQ	908	938	c	0.8710	1.3333	213

```
663  CCTATGCAAAATGATCCGGGGAATCGATTGG  693
     |:||||:|||| :||||||||||||||||||
938  CTTATGTAAAAAAATCCGGGGAATCGATTGG  908
```

SatDNA	From	To	Repbase	From	To	Dir	Sim	Pos/Mm:Ts	Score
TCsat15	1037	1102	Sola1-2_LMi	1445	1506	d	0.7846	2.0000	203

```
1037  AAAAATTTACTGGAGTGAGTTTAAAAAAAAATTTTTTTTCACCT-CTTTGAAGTTAAGTGTATTATT  1102
      ||||||||||||:|-||---|||| || | |||||||||:||:|-||:|||| || -|||||||:||
1445  AAAAATTTACTGAA-TG---TTAATAATACATTTTTTTTTACTTTCTCTGAACTTT-GTGTATTGTT  1506
```

TCsat16

SatDNA	From	To	Repbase	From	To	Dir	Sim	Pos/Mm:Ts	Score
TCsat16	148	184	DNAX-1_TCa	1481	1517	c	0.9189	1.0000	308

```
 148  TGTACAGGGTGCCGCATTTTGGATATCCGAATAGGAG  184
      ||||||||||||:|||||||||||:||||||||||:|
1517  TGTACAGGGTGCTGCATTTTGGATGTCCGAATAGGGG  1481
```

SatDNA	From	To	Repbase	From	To	Dir	Sim	Pos/Mm:Ts	Score
TCsat16	208	285	Dada-tV_PeFlu	7776	7849	d	0.7200	1.5833	234

```
 208 ATGCAATAAAAAATTGTACTACTGCTAAATCGAATCCAACTTGTTCAATTAACAGTAAATATATGTATC-   276
     ||:||||||-----|||||||:|::|||:|:||||:|||||| | | | ||:|||:| :|| | ||||:-
7776 ATACAATAA-----TGTACTATTATTAAGTTGAATTCAACTTTTACCAGTAGCAGCATGTAAAAGTATTA   7840

 277 TTGTCACAA 285
     ||||||:||
7841 TTGTCATAA 7849
```

TCsat19

SatDNA	From	To	Repbase	From	To	Dir	Sim	Pos/Mm:Ts	Score
TCsat19	13	77	MuDR-7_Cas	8094	8155	c	0.7778	1.7143	246

```
  13  GTTATT-TATCGAAAATTTGTATACTGATCTTGTATTTTTTTATTTTCAGTAGGTCTATTCTAAGA  77
      ||||||-|||| ||:||||||----|| |:||||||||:||| ||||: |:|||||:|| |||:||
8155  GTTATTGTATCTAAGATTTGT----TGCTTTTGTATTTCTTTTTTTTTTGCAGGTCCATGCTAGGA  8094
```

TCsat21

SatDNA	From	To	Repbase	From	To	Dir	Sim	Pos/Mm:Ts	Score
TCsat21	16	82	HipGal-6.520	280	353	c	0.7971	1.5000	220

```
16    AGTCGCAATAAATTTGT-----TAATTTGAAAATAAAAT--TTAATTCGC---GCTTCAAATTAATAAAA  75
      ||  ||||||:|||:|||-----|||||  |||||||||||--:|||||-:|---|:|||||--||||||||
353    AGGCGCAATGAATCTGTCAGATTAATTAGAAAATAAAATACCTAATT-ACTAAGTTTCAA--TAATAAAA  287

 76 ATGTACT 82
     |:||||
286 CTATACT 280
```

TCsat23

SatDNA	From	To	Repbase	From	To	Dir	Sim	Pos/Mm:Ts	Score
TCsat23	1	164	Rehavkus-1_TC	7923	8086	c	0.9939	1.0000	1441

```
   1 TTTAATATTATTAATAAAATGTTGACATATTTCAAACTAAGGGTTGGATGTCTTCCCAAACTTTTAGAGG   70
     ||||||||||||||||||||||||||||||||||||||||||||||||||||||||||||||||||||:|
8086 TTTAATATTATTAATAAAATGTTGACATATTTCAAACTAAGGGTTGGATGTCTTCCCAAACTTTTAGAAG   8017

  71 GTTGAAATTTTCAGGAAAGGTAGAGTATTTAATGGGCTTTCATGTGGAATGGGTTAAATGCGTCGACAAA   140
     ||||||||||||||||||||||||||||||||||||||||||||||||||||||||||||||||||||||
8016 GTTGAAATTTTCAGGAAAGGTAGAGTATTTAATGGGCTTTCATGTGGAATGGGTTAAATGCGTCGACAAA   7947

 141 AAGAAATAGTACTAGAAAAAAATA  164
     ||||||||||||||||||||||||
7946 AAGAAATAGTACTAGAAAAAAATA  7923
```

TCsat24

SatDNA	From	To	Repbase	From	To	Dir	Sim	Pos/Mm:Ts	Score
TCsat24	94	122	CerCot-5.769	495	523	d	0.9310	99.0000	226

```
 94  CCAGAGCGCATGCGCATGCGCGTCTGACC  122
     ||||||||| ||||||||||||| |||||
495  CCAGAGCGCCTGCGCATGCGCGTGTGACC  523
```

SatDNA	From	To	Repbase	From	To	Dir	Sim	Pos/Mm:Ts	Score
TCsat24	316	397	EnSpm-N2_DaCa	2493	2564	d	0.8108	1.6000	211

```
 316 TCACAAAGTCATG---ATTAAAATTTTAAAGTTTTTTTAACTTAATCGATAGACATGCGCGTAAGATTAA   382
     |:|||||||||||---||||||-|||||||--||||||||  |||  |:------||::|||  |||:||---
2493 TTACAAAGTCATGCAAATTAAA-TTTTAAA--TTTTTTAAATTATTT------CACACGCCTAAAAT---   2550

 383 AAAAATA-ATTTTTAT  397
     --|||||-||||||||
2551 --AAATATATTTTTAT  2564
```

TCsat26

SatDNA	From	To	Repbase	From	To	Dir	Sim	Pos/Mm:Ts	Score
TCsat26	68	260	Transib-3_DTa	3053	3197	d	0.7595	1.5625	241

```
  68 TTGTAACCATCACTGATTCTAAAAATAACTGAAAAGATTTAAAGTTAAAACGTTTTTATCTTCAAAGTAA   137
     ||||:|||||:|:|---||   |||||:|--|||||-----||----|||||-:|||||||:||:|||:-||
3053 TTGTGACCATTATT---TCAAAAAACA--TGAAA-----TA--------TAAAA-ATTTTTATTTTTAAAA-AA  3106

 138 ATATAGCTGTTAAATTTTCTCTTTTCTTTTTTTTGCAGTGTTCTGCTTAGTTTTCAAAAATTTTAATAAC   207
     |---|:||  |--||||||:|:||||:||||||||-------------|||||||:|||||||   ||:|
3107 A---AACTTT--AATTTTTTTTTTTTTTTTTTTTT--------------------- TAGTTTTTAAAAATTAAAACATA  3158

 208 AAAATGCTTTAATAAAAATTACTAATATAACACATAATTAAAACTAAAACAAA  260
     ||||------||||||-----||||-|:||  ||:|||||  |||--||||  |||
3159 AAAA------AATAAA-----CTAA-ACAAAACGTAATTTAAA--AAAAAAAA  3197
```

SatDNA	From	To	Repbase	From	To	Dir	Sim	Pos/Mm:Ts	Score
TCsat26	285	361	Gypsy-54_MT-I	1445	1515	c	0.7397	1.3333	230

```
 285 TTGTCAACTCGGCGCCTCCACCATTTTTAAT-TTTAGATATCCAACCAATTTACTTTCCTTACTTTTACT   353
     || ||||:||:::| ||:::| ||||||| |-|||:|||||:||||------|:|:||||||:|||||||
1515 TTCTCAATTCAATGACTTTGCGATTTTTATTCTTTGGATATTCAAC-----------ATTCTCCTTATTTTTACT  1452

 354 GTAAATGA 361
     |-||||||
1451 G-AAATGA 1445
```

TCsat27

SatDNA	From	To	Repbase	From	To	Dir	Sim	Pos/Mm:Ts	Score
TCsat27	21	57	Gypsy-22_DMel-I	4973	5009	c	0.8649	1.6667	234

```
  21  TTTTTCAAAGTTTTCTTGTCTGTTTTTGTCATATTTT  57
      ||||||:| ||| |||||||||:||||||||||||:|
5009  TTTTTCGATGTTATCTTGTCTGCTTTTGTCATATTCT  4973
```

TCsat28

SatDNA	From	To	Repbase	From	To	Dir	Sim	Pos/Mm:Ts	Score
TCsat28	99	167	MuDR-18_Cas	1384	1454	c	0.8000	5.0000	209

```
  99 GTACAAAAAATTTTGAACATTATTGTTTGGTAAAATTTTAAA---ATTAACAGAC-GGTTTT-TTATTTT   163
     ||||| |: ||||||||---||||||| | ||||| ||  ||---||||| |:||-||||||-|||||||
1454 GTACACAGTATTTTGAA---TATTGTTAGTTAAAAATTACAATGTATTAAGAAACTGGTTTTCTTATTTT   1388

 164 TTTT 167
     ||||
1387 TTTT 1384
```

SatDNA	From	To	Repbase	From	To	Dir	Sim	Pos/Mm:Ts	Score
TCsat28	216	342	EnSpm-14_RIr	5450	5579	c	0.7460	1.9167	226

```
 216 AATATACAGTTAAATACTTTAATGTG-TTTA---TTATTAAGTACTTGTAAGAAAAACCTTTTATTTTTG   281
     |||||||   ||||| |:|  :|||:||-||||---|||||------||::|| ||||| |||--||||||:
5579 AATATACTTTTAAAAATTGCAATATGATTTAAGCTTATT-----------TTACAATAAAAAGCTT--ATTTTTA  5518

 282 AC-TAA---GCTTAT-----GAAAAATTTATCACTTTTAAGTACAAAGAGTGTTTTATTTTAAAA-AACTT   342
     |  -|||---||||||----:||||::||||:--||| ||:|| ||||   |:||||||||||||-|||||
5517 AAATAAAAAGCTTATTTTTAAAAAGCTTATT--TTTAAAATAAAAAGCTTATTTTATTTTAAAATAACTT   5450
```

TCsat33

SatDNA	From	To	Repbase	From	To	Dir	Sim	Pos/Mm:Ts	Score
TCsat33	89	132	Mariner-5_DSuz	1411	1454	d	0.7955	1.8000	209

```
  89  TTATCTTTTTCCAGGTCGAAGCCCAAAAACTAATTATTTATGTC  132
      ||||:||||:| |||| :||:|||||||||:||||| |||| |||
1411  TTATTTTTTCCAAGGTAAAAACCCAAAAATTAATTCTTTAAGTC  1454
```

TCsat36

SatDNA	From	To	Repbase	From	To	Dir	Sim	Pos/Mm:Ts	Score
TCsat36	64	132	Helitron-N2_HMM	738	811	d	0.7500	2.1429	205

```
 64   CGATGTTATCCAATCAAGACAGCGGAATTTT--TAACTTGGTGTTTTATTTGA-ATTTTTC--AAGAATG  128
     ||||||||| || |:||:|:| || ||||||--| |:|||| :||||||||||-||| |||--||:|||
738   CGATGTTATACACTTAAAATATCGTAATTTTAATCATTTGGGATTTTATTTGATATTGTTCGCAAAAATT  807
```

```
129 GTTA 132
    :|||
808 ATTA 811
```

TCsat37

SatDNA	From	To	Repbase	From	To	Dir	Sim	Pos/Mm:Ts	Score
TCsat37	92	204	Mariner-32_RPr	1618	1729	c	0.7455	1.9091	202

```
  92  ATTAAATTTTAATTTTGAATTTAAATCAAATTTATTCAAGTACTCGAAATAA-----TTTGATTGCAA-T  155
      |||||| |||---|||:| ||:||||:|||||| ||:||:|--||:|| |||-----||| ||| |||-|
1729   ATTAAAGTTT---TTTAATTTCAAATTAAATTTTTTTAAAT--TCAAATTAAAGTTTTTTTATTTCAAAT  1665

 156  TAAGGGCCGGTTTACAGTAAGCGATTTAAA---TTTTAATTCCGAATTAAAG  204
      |||:--|:   |||| |---|||:| |||||---||||||||:|:||||||||
1664  TAAA--CTTTTTTAAA---AGCAAATTAAAGATTTTTAATTTCAAATTAAAG  1618
```

TCsat42

SatDNA	From	To	Repbase	From	To	Dir	Sim	Pos/Mm:Ts	Score
TCsat42	10	107	Gypsy-7_TCa-I	872	968	c	0.7113	1.8462	240

```
 10 ATTTTTTATTTTCTGAT--GTTGCTAATTTTTTTCTCGACTGTCGAAAGACAATTCGTTAAATTCTTATA   77
    |:|:|||||||||| :|--||| |||||||  |::||::|||:| ||||----||| ||: -||| ||||
968 ACTCTTTATTTTCTCGTACGTTTCTAATTTAGTCTTCAGCTGCCTAAAG--------TTCCTTGC-TTCATATA  904

 78 AC--TGTTCTAGTGCACATTTTGTTTCGAATT  107
    | --:|||:|| |:||||||||||:| |||||
903 AAAACGTTTTATTACACATTTTGTCTGGAATT  872
```

TCsat43

SatDNA	From	To	Repbase	From	To	Dir	Sim	Pos/Mm:Ts	Score
TCsat43	60	139	ARNOLDY2	424	501	c	0.7595	1.7500	209

```
 60 ATATCTTAAAAA------TTAACAGCAATCTACACAGCCTTGAAAAATCCTTAAAAAAATATTGCTAAACCT   125
    ||||:||||||  ----||||-|:||||:|-:|  |  ||||  |||||||:||||||||  :  |||-||||:||
501 ATATTTTAAAACCCTTTTAA-AACAATTT-TAAACCCTTTAAAAATCTTTAAAAAACCTTTG-TAAATCT   435

126 TTCGCTAAAACCCT  139
    ||---||||:||||
434 TT---TAAAGCCCT  424
```

TCsat45

SatDNA	From	To	Repbase	From	To	Dir	Sim	Pos/Mm:Ts	Score
TCsat45	190	227	COPIA3-I_MT	4552	4589	c	0.8421	1.2000	222

```
 190  GAAAATTTCTCCAACAAATAAATATTGTAGTGCATTTA  227
      |||:|||||||:||:||||||||  |||||:|:||||||
4589  GAAGATTTCTCTAATAAATAAATTTTGTAATACATTTA  4552
```

TCsat47

SatDNA	From	To	Repbase	From	To	Dir	Sim	Pos/Mm:Ts	Score
TCsat47	6	138	hAT-N20_ALy	185	314	d	0.7597	2.4444	211

```
  6 TTTTGTATTTTGAGAAATAACGTTTTG-AATATTTTATTCACT-TTATTCG----------TATTAATAAAGAAA 68
    ||||:||||  |----|||||::||||  -||||||||||-|||-|||||::-----||||||||  -----
185  TTTTATATTAT----AATAATATTTTTTAATATTTTATT-ACTATTATTTAAATTTTATTAATAT-----  244

 69  TTTCTAAATAAGTTGTTACATCTGTTCTTTATTTTTTATCAATCATTTTAATAATCTG--AT-TAATAAT  135
    |||:| ||||  ||:||||---|:|| ||||  ||||||:||| ||||| || |||| --||-|||||||
245 TTTTTTAATATTTTATTAC---TATTATTTAAATTTTATTAATAATTTTTATTATCTTTTATATAATAAT   311
```

```
136 AAT 138
    |||
312 AAT 314
```

TCsat48

SatDNA	From	To	Repbase	From	To	Dir	Sim	Pos/Mm:Ts	Score
TCsat48	57	136	Polinton-2_HM	130	211	d	0.7317	1.3846	216

```
 57  TAAGAAAAGAAAAGCTTAATCTTGTTTTACTCAAA--TGACACTATTTTTATAGAAAAAATGCTTAAAA-  123
    |||:||||:|||||  |||||:||-||||::|:|||--|:|:-|||  :  |||||:||||||||::  |||||-
130 TAAAAAAAAAAAAAGATTAATTTT-TTTTGTTTAAAGTTAAT-CTAGCGTTATAAAAAAAATATATAAAAA   197

124 -GCAAACGATTTTT  136
    -|||||:| |||||
198 AGCAAATGCTTTTT  211
```

TCsat49

SatDNA	From	To	Repbase	From	To	Dir	Sim	Pos/Mm:Ts	Score
TCsat49	47	132	Gypsy-6B_AlBr-I	4605	4687	c	0.8148	3.0000	240

```
  47  TATTGGTTTT---AATTAATTAA-----ATAATTAATTGACGTTGGAAGAGTTATTTTACATTTATAATC  108
      ||:|  |||||---|||||||||-----||||  -||||-||||||  ||||-------|||  |||:||||-
4687  TACTTGTTTTGTAAATTAATTAACTACTATAAG-AATT-ACGTTGTAAGA-------TACTTTTGTAAT-  4628

 109  GATATTTTCTTAAGACAAGTAAGC  132
      -||| |:||||||||| |||||||
4627  -ATAGTCTCTTAAGACTAGTAAGC  4605
```

SatDNA	From	To	Repbase	From	To	Dir	Sim	Pos/Mm:Ts	Score
TCsat49	175	261	hAT-26_DTa	334	406	c	0.7867	2.8000	219

```
175 TTAAAAAATATTAACAAAAAAACAGTTTTTGAGTGAACTTTACACTCGAATTTTGAACAAATAGTAAAAC   244
    ||||||||-|||||||||||||-------------| |||||:|||:| ||:| |  |||| ||  ||:|
406 TTAAAAAA-ATTAACAAAAAAA-------------ATCTTTATACTTGCATCTGGTTCAAAAAGATAAGC   351

245 AAAAATTTATTTTTTTT  261
    ||||||  ||||:|||||
350 AAAAATATATTCTTTTT  334
```

TCsat50

SatDNA	From	To	Repbase	From	To	Dir	Sim	Pos/Mm:Ts	Score
TCsat50	12	105	Gypsy-45_PAb-I	3823	3921	d	0.8065	1.8000	215

```
  12  TTTGACAAACTTACTATTTAAGAAACA-----TAAATTAA----TCACAATCTGTTATAACTTAAAAAAA  72
      |||:||:|||  |||||||----|||||-----:|||||:|----|||-|||||-|  ||||----||||||
3823  TTTAACGAACATACTATT----AAACAATTGTCAAATTGACATATCA-AATCT-TGATAA----AAAAAA  3882

  73  ATTAAAAAT-GTCCTT----AGGTAACAAA-TTATTTAC  105
       ||||||||-|||  ||----||:|||||||-||||||||
3883  TTTAAAAATAGTCATTGGAAAGATAACAAAATTATTTAC  3921
```

TCsat51

SatDNA	From	To	Repbase	From	To	Dir	Sim	Pos/Mm:Ts	Score
TCsat51	59	143	Gypsy-671_AA-I	292	373	d	0.7619	3.0000	211

```
 59 ATAAAAACTTG-GTAAAATTGACTTCGCTTATTTAT-TCAGTGAT-ATCTTCGTAATTACTAAGTTTATG   125
    |||||| | |:-|||:||||:|----|||| |||||-| ||||||-|||   ||:|||--|||||  |||
292 ATAAAAACCGTAAGTAGAATTAA-----GCTTTTTTATATAAGTGATTATCAGAGTGATT--TAAGTGAATG   355
```

```
126 ATCAAAATAATAACATAT  143
    ||||||||||:|| ||||
356 ATCAAAATAACAAAATAT  373
```

SatDNA	From	To	Repbase	From	To	Dir	Sim	Pos/Mm:Ts	Score
TCsat51	170	243	MuDRx-3_TV	699	768	c	0.7639	2.0000	221

```
170 AGAAAAGCTTAATTTTAACC-CAATTTTTAGTTTTATTTTATTAATACAAATTTTCGAATTTATTATTTA     238
    |||||||||| |||||  :|-|||||||||:|||:||||:| | -| :|||:||||----|||:||| ||
768 AGAAAAGCTTTATTTTTTTCTCAATTTTTAATTTCATTTCAGTC-TCTAAACTTTC --------TTACTATGTA     704

239 AAAAC 243
    |||||
    AAAAC 699
```

SatDNA	From	To	Repbase	From	To	Dir	Sim	Pos/Mm:Ts	Score
TCsat51	255	335	Helitron-N60B_CGi	3066	3153	d	0.7765	2.6000	223

```
 255 AGCT-TCACTTATTTA----TCAACCGATATCTTAAAAAT-TACTCAATTTATGGCTAAACTAA---TTA    315
     ||||-| |||| ||||----||| |||||||-||||||||-| :|||:||||| |:||:| |||--- ||
3066 AGCTGTAACTTTTTTACCTTTCATCCGATAT-TTAAAAATCTTTTCAGTTTATTGTTAGAATAAAGAGTA    3134

 316 CAGATTTAAAAAATATGATC  335
     ||-||||||||| ||||:||
3135 CA-ATTTAAAAATTATGGTC  3153
```

TCsat52

SatDNA	From	To	Repbase	From	To	Dir	Sim	Pos/Mm:Ts	Score
TCsat52	4	35	Academ-10_LMi	666	697	c	0.9062	1.5000	225

```
  4  TTTGTAGAGAATTTTATTGTCTTTAATTTTGT  35
     ||||||||  ||||||:|:|||||||||||||
697  TTTGTAGACAATTTTGTCGTCTTTAATTTTGT  666
```

TCsat53

SatDNA	From	To	Repbase	From	To	Dir	Sim	Pos/Mm:Ts	Score
TCsat53	9	140	MuDR-2_GAr	3549	3679	c	0.7405	1.9231	233

```
   9 TTTTTTCAGTTATATTTTATTCTTTTTTAGTTTCTCGGGCATTTA--TTGTTTTATTTTCTC--TTAATC    74
     |:|||||| ||| |||| | |:||||:| :||||------|||||--||: |||-|||||||--|| ||:
3679 TCTTTTCATTTAAATTTAAATTTTTTCTCATTTC----------- ATTTAAATTAATTT-TTTTCTCATTTCATT   3617

  75 TCGGTT-ATTTTACTTTGCAGTTTTTAAGTTAAACTTCTCAT--GGTTTAGACT--ATTTTTTTCTCATT    139
     |  ::||-||||| ||||-||--|||||| |||||:||:||||-- :||||:|:|-- ||||||||||||
3616 TAAATTTATTTTTCTTT-CA--TTTTAATTTAAATTTTTCATTTCATTTAAATTTANTTTTTTTCTCATT    3550

 140 T 140
     |
3549 T 3549
```

TCsat54

SatDNA	From	To	Repbase	From	To	Dir	Sim	Pos/Mm:Ts	Score
TCsat54	12	68	HARB-N1_TC	819	876	c	0.7931	2.0000	209

```
 12  ACAAATA--TTTGTATTAAATAATGAGAAATAGGTGAAAATCAAGCGAATAAAAATGAA  68
     |:|||||--||| ||||:||||||:| |||||::|-||||||||| ||| |||| ||||
876  ATAAATAATTTTTTATTGAATAATAATAAATAAAT-AAAATCAAGAGAAAAAAATTGAA  819
```

SatDNA	From	To	Repbase	From	To	Dir	Sim	Pos/Mm:Ts	Score
TCsat54	166	264	Gypsy-122_NS-LTR	147	237	c	0.7660	1.7778	232

```
166 CGTACAATTTTTTTATTATTCCTTTGGAAATTTTGCAAAACAATCTTGTATTGAGTTATTGTAGAAA---   232
    |||||| | ||||||| |||::||:----||||||:||||||| |||:-|||:|:|-|||----|||---
237 CGTACATTATTTTTATGATTTTTTC----ATTTTGTAAAACAAACTTA-ATTAAAT-ATT----AAACGC   178

233 AAATAACGACAGTTTTTGTATTTTTTTTTTAA  264
    ||||:-||||| |||||:|||||||| ||||
177 AAATG-CGACATATTTTTATATTTTTTATTAA  147
```

SatDNA	From	To	Repbase	From	To	Dir	Sim	Pos/Mm:Ts	Score
TCsat54	265	309	Gypsy-27_DWil-I	295	333	c	0.8500	2.5000	201

```
265  ATTTTGACTTTTATTTTACCAAAAAATATTGTTTCTTATTTAAGA  309
     |||||||:|| |||||||------||| |:| |||||||||||||
333  ATTTTGATTTGTATTTTA------AATTTCGATTCTTATTTAAGA  295
```

TCsat55

SatDNA	From	To	Repbase	From	To	Dir	Sim	Pos/Mm:Ts	Score
TCsat55	146	309	DNA-1_CQ	1390	1543	d	0.7421	1.6471	275

```
 146 AAAATTT--AAGATT-CTAAAATT-CAAAATATGTACCCAAAGTTTTCGAAACTTTAGACTTGAACATGT   211
     |||||||--||:|||-||||||||-| |||| |---|::|||--|||| |||:||----|||:||: | |
1390 AAAATTTCTAAAATTTCTAAAATTTCTAAATTT---CTTAAA--TTTCTAAATTT---------CTTAAATTTCT 1450

 212 GAACTT-TGAAACTTCTAAACTTCTAAATAACTAAAACTTGAAGATCTAAAGTTTC-AAAGTTGTTCTAA   279
     :||:||-|:|||:|||||||:||||||||   ||  |||:||------|||||:||||-|||:||--|||||
1451 AAATTTCTAAAATTTCTAAATTTCTAAATTTCTTAAATTT------------CTAAAATTTCTAAAATT--TCTAA  1512

 280 G-TTTC-AAGATTAGGTACAATTTCTAAGATT  309
     :-||||-||:|||-  || |||||||||:|||
1513 AATTTCTAAAATT-TCTAAAATTTCTAAAATT  1543
```

TCsat56

SatDNA	From	To	Repbase	From	To	Dir	Sim	Pos/Mm:Ts	Score
TCsat56	191	244	Harbinger-21_OS	1503	1558	c	0.8182	1.4000	258

```
 191  CATTTAAATTATTTTACCAAGCATTTAC----AATAATTTACTCAGAAGATGTCTATT  244
      |||||:|||||||:  ||||||||:| ||----||||-||||-|||:|||||||:||||
1558  CATTTGAATTATTCAACCAAGCACTGACCTAAAATA-TTTA-TCAAAAGATGTTTATT  1503
```

B)

Repeat Class	Fragments	Length (bp)
**Transposable Element**	37	2955
**DNA transposon**	27	2267
Academ	2	60
Dada	1	78
EnSpm/CACTA	2	209
Harbinger	2	111
Helitron	2	150
Mariner/Tc1	2	157
MuDR	6	584
Polinton	1	80
Sola	1	66
Sola1	1	66
Transib	2	224
hAT	3	280
piggyBac	1	67
**LTR Retrotransposon**	9	659
Copia	1	38
Gypsy	8	621
**Non-LTR Retrotransposon**	1	29
L1	1	29
**Total**	**37**	**2955**

C)

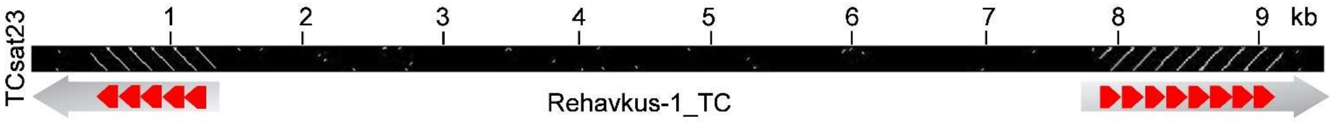

Figure S2. Similarities between *T. castaneum* low-copy satDNAs and Repbase entries (release 27.09) accessed by the software CENSOR. **A)** Alignments of partial sequence segments between *T. castaneum* low-copy satDNAs and Repbase-deposited repetitive elements. **B)** A report classifying all repetitive elements in Repbase with which the *T. castaneum* low-copy satDNAs share partial similarity. **C)** A dot-plot sequence comparison showing TCsat23 monomers (red arrows) repeated in the inverted termini (grey arrows) of Rehavkus-1_TC DNA transposon.
