# Supplementary material for: The Low-Copy-Number Satellite DNAs of the Model Beetle Tribolium castaneum
Source: Genes (Basel). 2023 Apr 28;14(5):999. doi: 10.3390/genes14050999 (PMC10218199; doi:10.3390/genes14050999)
Supplement: Supplementary file 1 [file genes-14-00999-s001.zip › Supplementary materials/Figure S3.pdf]

A) TCsat37

TCsat37 subfamilies' identity (%) matrix

	TCSat37-330	TCSat37-270	TCSat37-211
TCSat37-330		64.207%	65.254%
TCSat37-270	64.207%		70.632%
TCSat37-211	65.254%	70.632%	

```
                       10        20        30        40        50        60        70        80        90       100
               ....|....|....|....|....|....|....|....|....|....|....|....|....|....|....|....|....|....|....|....|
TCsat37-330    AAAATTGGGGCGCGAAT----TAAACTTTAATTTCGAAACGAAACAAATTAACC------TCGAACTTGAATGCA------------------------- 65
TCsat37-270    --.T.C.AA.....G.CCGT.T..T....A..TA..CAA.TT.....G..ATCGAACT.TA.TT.C...ATG.GACAAATTTACCTTGAACTTGAATG 98
TCsat37-211    --.T.C.AA.......CCGT.T..G....A..TA..CAA.TTT...G..AA------.TA.C.....ATA.------------------------- 67

                      110       120       130       140       150       160       170       180       190       200
               ....|....|....|....|....|....|....|....|....|....|....|....|....|....|....|....|....|....|....|....|
TCsat37-330    --TTCAATTTAGCGGCCGGTTTTATTTATTTATTAAATTTTAATTTACAATTTAAATCAAATTTATGCACGTACTCGAAATAATTTGATTGCAATTAAGA 163
TCsat37-270    CACG..C...CA.........A.......................TG........G.......T..A....T...................T..G 198
TCsat37-211    --.G..C...CA.........A...................-...TG..................T..A................A.......CCG.G 164

                      210       220       230       240       250       260       270       280       290       300
               ....|....|....|....|....|....|....|....|....|....|....|....|....|....|....|....|....|....|....|....|
TCsat37-330    GCCGGTTTACAGCAAACGA--ATTAAATTTAATTCCGA-TTAAAGAGTAATTTGTGCGTGTGCGTCTGTTTGTTAAATTAATTATTAAATAAATTATTAA 260
TCsat37-270    ............T.....TT..A..T..........A.......A..G.....TTTC..TT-..AA..CAC--------------------------- 270
TCsat37-211    ...............G..CTGG.------------------------........TTTC..TT-..AA..CA---------------------------- 211

                      310       320       330       340       350       360       370
               ....|....|....|....|....|....|....|....|....|....|....|....|....|....|..
TCsat37-330    ACTGCGACCGCAACCGCGACCGCGACACGAATTAATTTAAGTAATTCTGTGCCGTGCCGTACACTTTTATTA 332
TCsat37-270    ------------------------------------------------------------------------ 270
TCsat37-211    ------------------------------------------------------------------------ 211
```

B) TCsat38

TCsat38 dimeric HOR

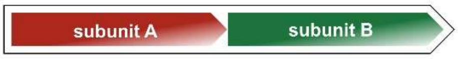


```
                          10        20        30        40        50        60        70        80        90       100
                  ....|....|....|....|....|....|....|....|....|....|....|....|....|....|....|....|....|....|....|....|
TCsat38subunitA   ATACCAAATCAAG-ATTTTTTTGGCAAAGTTCCAATCAATACTTTTGAACGAAATTTTGCAAACTAATAGACATTAAACAAAGCTATAATGGTACAAAAG 99
TCsat38subunitB   -.......-..T.A..........C........C...T..T........AC.......G...T.G..G.......G.G....T......C........T 98

                         110       120       130       140       150       160       170       180
                  ....|....|....|....|....|....|....|....|....|....|....|....|....|....|....|....|....|....|.
TCsat38subunitA   CTCGTTTTRAAAGACAGTTCTGTTCAGCAGATATAATTTTTTGAATTTAA-TTGAGGCAAACTTAGGTTTTTGCTATCTGA 179
TCsat38subunitB   ..TA......T.......CGA....G.A.....CG.A.............G......T...A.C.A.......T....... 179
```

C) TCsat18

TCsat18 monomer variants' identity (%) matrix

	TCSat18-73	TCSat18-63	TCSat18-82
TCSat18-73		82.192%	75.610%
TCSat18-63	82.192%		64.634%
TCSat18-82	75.610%	64.634%	

```
                   10        20        30        40        50        60        70        80
              ....|....|....|....|....|....|....|....|....|....|....|....|....|....|....|....|..
TCsat18-73    TTGGCAACGTGTTTTAAACACGTGTCCGCGCCATTTTTTAAATTTAATTACCGATTTCGTGCGATTTTTTAGG--------- 73
TCsat18-63    ...............G............T........T.............----------........--------- 63
TCsat18-82    .........................C......G..............G.C..GTTTTAAACACGTGTCCGCGCCATTTTTTAA 82
```

D) TCsat15-1277 variant

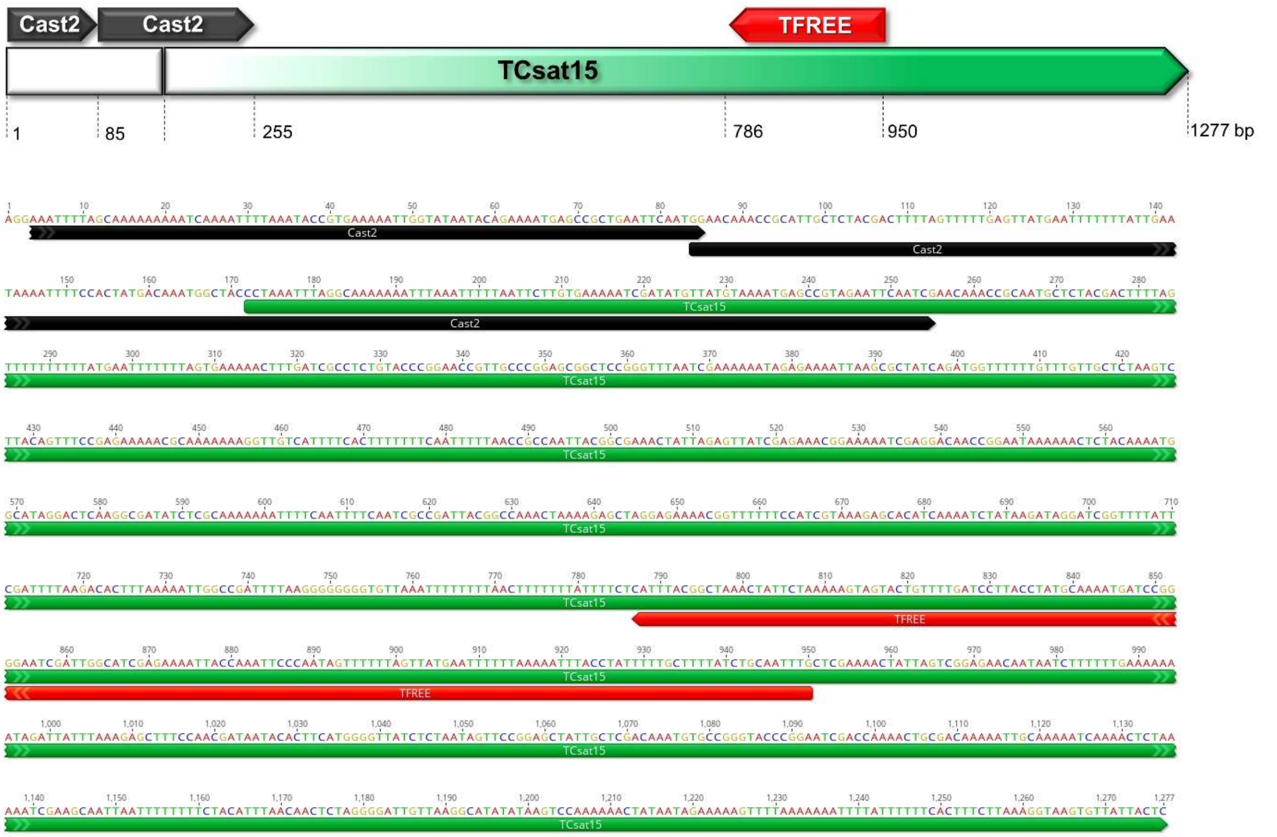


```
                  10        20        30        40        50        60        70        80        90       100
              ....|....|....|....|....|....|....|....|....|....|....|....|....|....|....|....|....|....|....|....|
Cast2         GGAACAAACCGCATTGCTCTACGACTTTTAGTTTTTGAGTTATGAATTTTTTTTTTTGAATAAAATTTTCCACTATAGATATTGCCTATCTTAATTTTTAG 100
TCsat15-1277  .....................................................A.....................GACA.A..G...C.C...A...AG. 100

                 110       120       130       140       150       160       170
              ....|....|....|....|....|....|....|....|....|....|....|....|....|....|..
Cast2         CAAAAAACTTTAAAAGTTTACATCTTGTTAAAAGTTGGTTTTATACGCAAAATGAGCCGCTGAATTCAATGA 172
TCsat15-1277  .......A......TT....AT......G....A.C.A.A.GT..T.T...........TA.........CG 172
```

```
                  10        20        30        40        50        60        70        80        90       100
              ....|....|....|....|....|....|....|....|....|....|....|....|....|....|....|....|....|....|....|....|
TFREE         CACTTACGGCTATACTATATTCGAAACAGTGGAACGTTTCGACCCTTGCCGATGCAAAATGATCCAGGTAATCGAT-GAAGTCACAAAAATCGCAGTATT 99
TCsat15-1277  ..T.........A.....TC.AA...GTA-.T.CT....T..T....A..T..............G..G.......T.GCA..GAG.....TA.CAA... 99

                 110       120       130       140       150       160       170
              ....|....|....|....|....|....|....|....|....|....|....|....|....|....|....|
TFREE         CCTAATAGTTTTTGAATTATGAACTATTTTTTACTTTTTATAAATTT--------TTGATTTTATTTATTTTTTC 166
TCsat15-1277  ..C..........T.G.......T.----------.....A......ACCTATTT...C......C.GCAA...G 164
```

Figure S3. Specifics of satDNAs TCsat37, TCsat38, TCsat18, and TCsat15. **A)** Identity matrix and alignment of the consensus sequences of the three TCsat37 subfamilies TCsat37-330, TCsat37-270, and TCsat37-211. **B)** Structural organization of satDNA TCsat38 dimeric higher-order repeat (HOR) and alignment of the consensus sequences of subunits A and B. **C)** Identity matrix and alignment of the consensus sequences of the three TCsat18 monomer variants TCsat18-63, TCsat18-73, and TCsat18-82. A segment duplicated in the variant TCsat18-82 is highlighted in red. **D)** Schematic presentation of the structural organization of the TCsat15-1277 monomer variant. Black arrows indicate segments that have similarity to satDNA Cast2, while the red arrow marks the position of the segment that shares similarity with the satDNA TFREE from the sibling species *Tribolium freemani*. The alignment between TCsat15-1277 and TFREE consensus sequences is shown at the bottom of the panel. In all alignments, identities between the compared sequences are indicated by dots (.), and dashes (-) indicate alignment gaps.
