# Supplementary material for: The Low-Copy-Number Satellite DNAs of the Model Beetle Tribolium castaneum
Source: Genes (Basel). 2023 Apr 28;14(5):999. doi: 10.3390/genes14050999 (PMC10218199; doi:10.3390/genes14050999)
Supplement: Supplementary file 1 [file genes-14-00999-s001.zip › Supplementary materials/Figure S4.pdf]

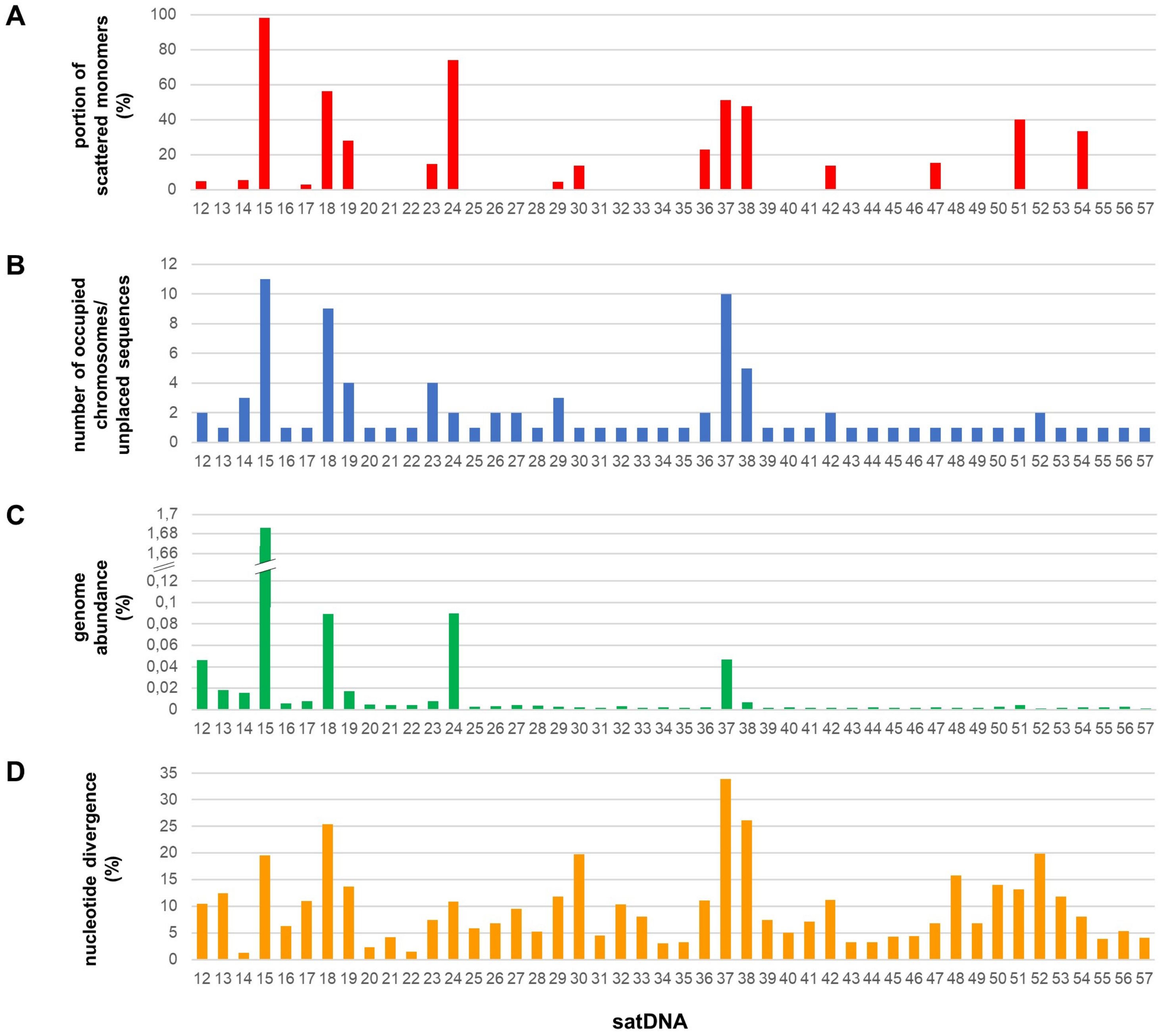

Figure S4. Comparative analyses of 46 *T. castaneum* satDNAs identified in this work with respect to: **A)** the proportion of scattered repeats among annotated monomer copies, **B)** the number of chromosomes/unplaced sequences at which a satDNA is annotated, **C)** genome abundance, **D)** nucleotide divergence calculated for annotated monomers. The charts are based on the data presented in the Table S3.
