# Supplementary material for: The Low-Copy-Number Satellite DNAs of the Model Beetle Tribolium castaneum
Source: Genes (Basel). 2023 Apr 28;14(5):999. doi: 10.3390/genes14050999 (PMC10218199; doi:10.3390/genes14050999)
Supplement: Supplementary file 1 [file genes-14-00999-s001.zip › Supplementary materials/Figure S5.pdf]

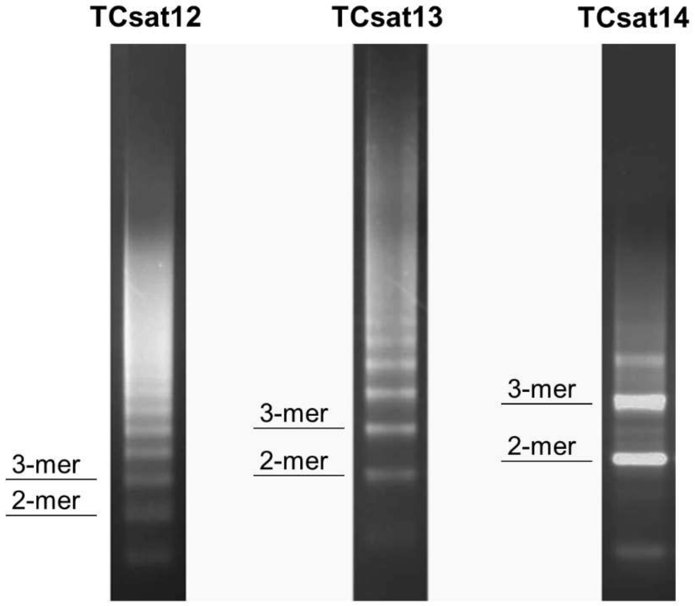

Figure S5. Gel electrophoresis of PCR-amplified fragments using specific primers for satDNA TCsat12, TCsat13, and TCsat14. Fragments corresponding to dimers and trimers from each PCR amplification were extracted from the gel and used for further cloning.
