# Supplementary material for: The Low-Copy-Number Satellite DNAs of the Model Beetle Tribolium castaneum
Source: Genes (Basel). 2023 Apr 28;14(5):999. doi: 10.3390/genes14050999 (PMC10218199; doi:10.3390/genes14050999)
Supplement: Supplementary file 1 [file genes-14-00999-s001.zip › Supplementary materials/Figure S6.pdf]

A) TCsat12

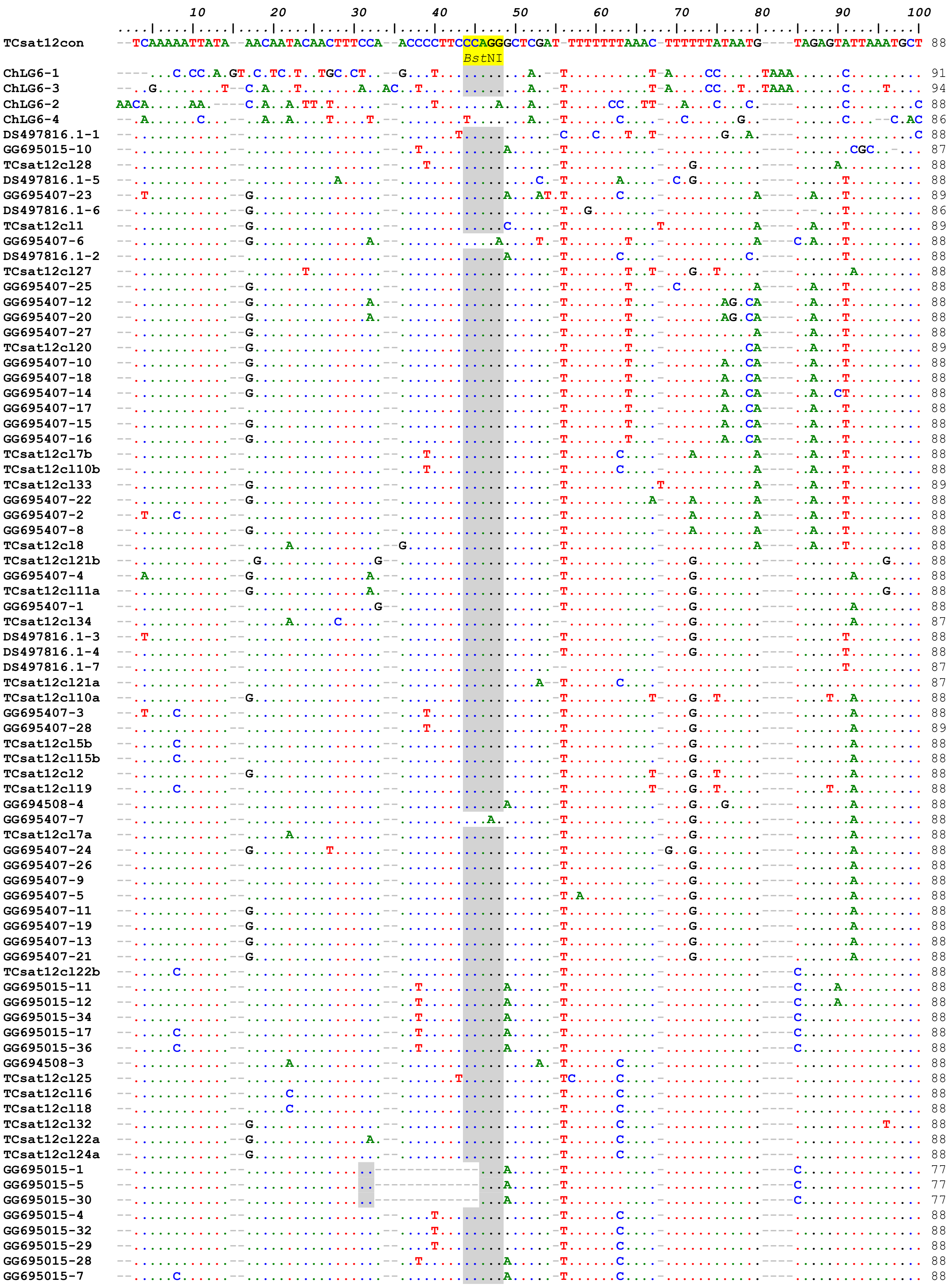
10 20 30 40 50 60 70 80 90 100
TCsat12con --TCAAAAATTATA--AACAATACAACTTTCCA--ACCCCTTCCCAGGGCTCGAT-TTTTTTTAAAC-TTTTTTATAATG---TAGAGTATTAAATGCT 88
BstNI
ChLG6-1
ChLG6-3
ChLG6-2
ChLG6-4
DS497816.1-1
GG695015-10
TCsat12cl28
DS497816.1-5
GG695407-23
DS497816.1-6
TCsat12cl1
GG695407-6
DS497816.1-2
TCsat12cl27
GG695407-25
GG695407-12
GG695407-20
GG695407-27
TCsat12cl20
GG695407-10
GG695407-18
GG695407-14
GG695407-17
GG695407-15
GG695407-16
TCsat12cl7b
TCsat12cl10b
TCsat12cl33
GG695407-22
GG695407-2
GG695407-8
TCsat12cl8
TCsat12cl21b
GG695407-4
TCsat12cl11a
GG695407-1
TCsat12cl34
DS497816.1-3
DS497816.1-4
DS497816.1-7
TCsat12cl21a
TCsat12cl10a
GG695407-3
GG695407-28
TCsat12cl5b
TCsat12cl15b
TCsat12cl2
TCsat12cl19
GG694508-4
GG695407-7
TCsat12cl7a
GG695407-24
GG695407-26
GG695407-9
GG695407-5
GG695407-11
GG695407-19
GG695407-13
GG695407-21
TCsat12cl22b
GG695015-11
GG695015-12
GG695015-34
GG695015-17
GG695015-36
GG694508-3
TCsat12cl25
TCsat12cl16
TCsat12cl18
TCsat12cl32
TCsat12cl22a
TCsat12cl24a
GG695015-1
GG695015-5
GG695015-30
GG695015-4
GG695015-32
GG695015-29
GG695015-28
GG695015-7

```
GG695015-31   --.....C......--.................--...................-TC.....C....-............----................ 88
GG695015-3    --............--.................--...................-TC.....C....-............----................ 88
GG695015-2    --............--.................--...................-TC.....C....-............----................ 88
GG695015-14   --.....C......--.................--...................-TC.....C....-............----................ 88
GG695015-24   --............--.................--..T..........A.....-T......C....-............----..........T..... 88
GG695015-25   --............--.................--.............A.....-T......C....-............----..........G..... 88
TCsat12cl30b  --............--.......T.........--..A................-T......C....-............----..........T..... 88
GG695015-38   --............--.................--..T................-T......C....-............----..........T..... 88
GG695015-8    --.....C......--.................--..A................-T......C....-............----..........T..... 88
GG695015-9    --............--.................--..A................-T......C....-............----................ 88
GG695015-39   --............--.................--..AT...............-T......C....-............----................ 88
TCsat12cl11b  --............--.................--.A.................-T......C....-............----................ 88
GG695015-15   --............--.................--....T..............-T......C....-............----................ 88
GG695015-16   --............--.................--....T..............-T......C....-............----................ 88
TCsat12cl5a   --............--.................--...................-T......C....-............----................ 88
TCsat12cl15a  --............--.................--...................-T......C....-............----................ 88
GG695015-13   --............--.................--..T..........A.....-T......C....-............----C............... 88
GG695015-37   --............--.................--.............A.....-T......C....-............----................ 88
GG695015-19   --............--.................--.............A.....-T......C....-............----................ 88
GG695015-35   --............--.................--.............A.....-T......C....-............----................ 88
TCsat12cl30a  --............--.................--.............A.....-T......C....-............----................ 88
TCsat12cl24b  --............--.................--.............A.....-T......C....-............----C............... 88
GG694508-1    --............--...............T.--...................-TC.....C....-............----................ 88
GG694508-2    --.....C......--.................--...................-TC.....C....-............----................ 88
GG695015-33   --............--.................--...........A.......-TC.....C....-............----................ 88
GG695015-22   --.A..........--.................--...................-TC.....C....-............----C............... 88
GG695015-23   --............--.................--...................-TC.....C....-............----C............... 88
GG695015-26   --............--.................--...................-TC.....C....-............----C............... 88
GG695015-27   --............--.................--...................-TC.....C....-............----C............... 88
GG695015-6    --............--.................--...................-TC.....C....-............----C............... 88
GG695015-18   --............--.................--...................-TC.....C....-............----C............... 88
GG695015-20   --............--.................--...................-TC.....C....-............----C............... 88
GG695015-21   --............--.................--...................-TC.....C....-............----C............... 88
```

```
                   110       120       130       140       150       160       170
              ....|....|....|....|....|....|....|....|....|....|....|....|....|....|.
TCsat12con    CTTTCGACCTGCCGTAACAAGAAAGTGGCACATTGTCGATAACACATAG----AAAAAAAATAAAAACCA 154

ChLG6-1       ....T.....CTA....G..A..C..A.....C...T..A.........----.......T......AAT- 157
ChLG6-3       ....T......-----------------........T..A..-.T....---A......TGA.......-- 142
ChLG6-2       ............AC..G..GA.....CT........T....T.......--AA.......T........T- 156
ChLG6-4       ....TC....A.AC.....GA......T........T....T.......----.......T........T- 152
DS497816.1-1  ....................A......A..............G.T....-----.........C.....GT 154
GG695015-10   .........................AAA.....................----.................- 153
TCsat12cl28   .................T..A.........................G.A----.................- 154
DS497816.1-5  .................................................----.........G.....A.- 154
GG695407-23   .................................................----.................- 155
DS497816.1-6  ............T....................................----........AG.......- 152
TCsat12cl1    ....A............................................----.................- 155
GG695407-6    ...........T.T...........C.......................----.................- 154
DS497816.1-2  ............................T.......T.T..........----.........G.......- 154
TCsat12cl27   ...G.........T...........C......C................----.................- 154
GG695407-25   .............T...........C....A..................----.................- 154
GG695407-12   .............T...................................----.................- 154
GG695407-20   .............T...................................----.................- 154
GG695407-27   .............T......A....C.......................----.................- 154
TCsat12cl20   .............T...........C.......................----.................- 155
GG695407-10   .............T...........C.......................----.................- 154
GG695407-18   .............T...........C.......................----.................- 154
GG695407-14   .............T...................................----.................- 154
GG695407-17   .............T...................................----.................- 154
GG695407-15   .............T...................................----.................- 154
GG695407-16   .............T...................................----.................- 154
TCsat12cl7b   .............T...................................---A.................- 155
TCsat12cl10b  ...........A.T...........C.......................----.................- 154
TCsat12cl33   ............TT...........C.......................---A.............G...- 156
GG695407-22   .............T...................................----.................- 154
GG695407-2    ...........A.T...........C.......................----.................- 154
GG695407-8    ...........A.T...........C.......................----.................- 154
TCsat12cl8    ....A............................................----.................- 154
TCsat12cl21b  .................................................----.................- 154
GG695407-4    .................................................----.................- 154
TCsat12cl11a  .................................................----.................- 154
GG695407-1    .................A.G.............................----.................- 154
TCsat12cl34   ......................G..........................----.................- 153
DS497816.1-3  .............T...........C.......................----.................- 154
DS497816.1-4  ......................G..........................----....-----........- 149
DS497816.1-7  .................................................----................T- 153
TCsat12cl21a  ............TT...........C.......................----.................- 153
TCsat12cl10a  ...........A.T...........C....................A..----.................- 154
GG695407-3    .............T...........C....T..................----.................- 154
GG695407-28   .............T...........C....T.................A----.................- 155
TCsat12cl5b   ............TT...........C.......................----.................- 154
TCsat12cl15b  ............TT...........C.......................----.................- 154
TCsat12cl2    ............TT...........C.......................----.................- 154
TCsat12cl19   ............TT...........C.......................----.................- 154
```

```
GG694508-4     .........................C.......................-----................- 153
GG695407-7     ........T....T...........C....T..................----.................- 154
TCsat12cl7a    ........T....T...........C....T..................----.................- 154
GG695407-24    ........T....T...........C....T..................----.................- 154
GG695407-26    ............GT...........C....T..................----.................- 154
GG695407-9     .........................C....T..................----.................- 154
GG695407-5     .............T...........C....T..................----.................- 154
GG695407-11    .............T...........C....T..................----.................- 154
GG695407-19    .............T...........C....T..................----.................- 154
GG695407-13    .............T.........G.C....T..................----.................- 154
GG695407-21    .............T.........G.C....T..................----.................- 154
TCsat12cl22b   ...------.....................................G.-----.................- 147
GG695015-11    ...------........T...............................---A................- 149
GG695015-12    ...------........................................---A................- 149
GG695015-34    T..------........T...............................---A................- 149
GG695015-17    ...------........T...............................---A................- 149
GG695015-36    ...------........A...............................---A................- 149
GG694508-3     ...------........................................---A................- 149
TCsat12cl25    ....T............T...............................--AA.............G...- 156
TCsat12cl16    ........................A........................----.................- 154
TCsat12cl18    ........................A........................----.................- 154
TCsat12cl32    T................................................----.................- 154
TCsat12cl22a   .............................................G...----.................- 154
TCsat12cl24a   .................................................----.................- 154
GG695015-1     ..........................................T....T.--AA.................- 145
GG695015-5     ..........................................T....T.--AA.................- 145
GG695015-30    ..........................................T....T.--AA.................- 145
GG695015-4     ..........................................T....T.--AA.................- 156
GG695015-32    ..........................................T....T.--AA.................- 156
GG695015-29    ..........................................T....T.A-AA.................- 157
GG695015-28    ..........................................T....T.--AA.................- 156
GG695015-7     ..........................................T....T.A-AA.................- 157
GG695015-31    ...------.................................T....T.--AAT................- 150
GG695015-3     ...------.................................T....T.--AAT................- 150
GG695015-2     ..........................................T....T.--AA.................- 156
GG695015-14    ..........................................T....T.A-AA.................- 157
GG695015-24    .........................................C.......----.................- 154
GG695015-25    .........................................C.......----.................- 154
TCsat12cl30b   .................................................----.................- 154
GG695015-38    .................................................----.................- 154
GG695015-8     .................................................----.................- 154
GG695015-9     .................................................---A................- 155
GG695015-39    .................................................----.................- 154
TCsat12cl11b   ..........A......................................----.........T.......- 154
GG695015-15    .................................................---A................- 155
GG695015-16    .................................................----.................- 154
TCsat12cl5a    ..........A......................................---A................- 155
TCsat12cl15a   ..........A......................................---A................- 155
GG695015-13    ...------........................................----.................- 148
GG695015-37    ...------........................................---A................- 149
GG695015-19    .................................................---A................- 155
GG695015-35    .................................................----.................- 154
TCsat12cl30a   .................................................----.................- 154
TCsat12cl24b   ...------........................................----.................- 148
GG694508-1     .................................................----.................- 154
GG694508-2     .................................................----.................- 154
GG695015-33    ....T............................................---A................- 155
GG695015-22    ...------........T.......................C.......----.................- 148
GG695015-23    ...------........T.......................C.......----.................- 148
GG695015-26    ...------........T.......................C.......----.................- 148
GG695015-27    ...------........T.......................C.......----.................- 148
GG695015-6     ...------........................................----.................- 148
GG695015-18    ...------........T...............................---A................- 149
GG695015-20    ...------........T...............................---A................- 149
GG695015-21    ...------........T...............................---A................- 149
```

B) TCsat13

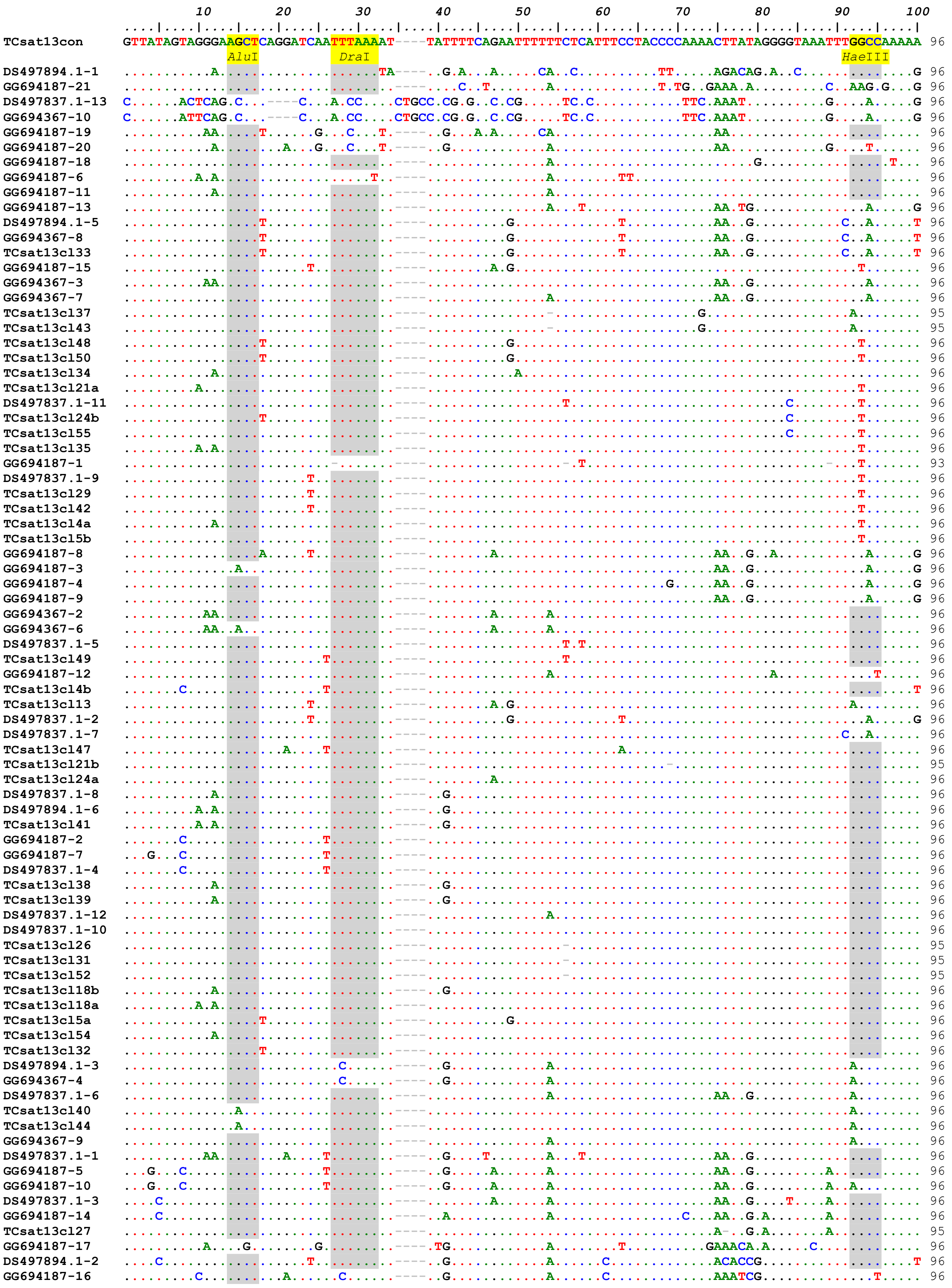
TCsat13con
GTTATAGTAGGGAAGCTCAGGATCAATTTAAAAT----TATTTTCAGAATTTTTTCTCATTTCCTACCCCAAAACTTATAGGGGTAAATTTGGCCAAAAA 96
AluI
DraI
HaeIII

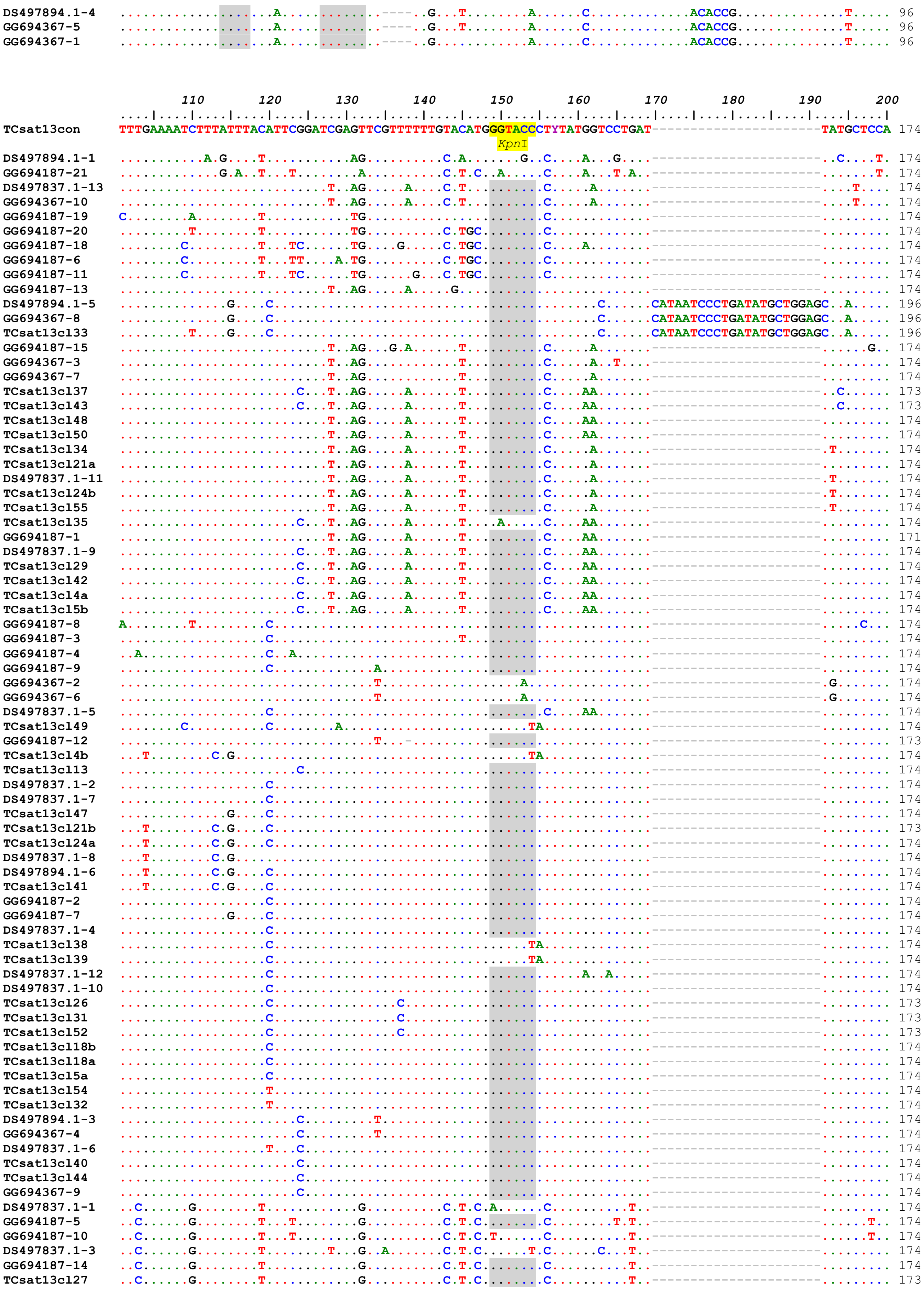

DS497894.1-4 96
GG694367-5 96
GG694367-1 96
110 120 130 140 150 160 170 180 190 200
TCsat13con TTTGAAAATCTTTATTTACATTCGGATCGAGTTCGTTTTTTGTACATGGGTACCCTYTATGGTCCTGAT TATGCTCCA 174
KpnI
DS497894.1-1 174
GG694187-21 174
DS497837.1-13 174
GG694367-10 174
GG694187-19 174
GG694187-20 174
GG694187-18 174
GG694187-6 174
GG694187-11 174
GG694187-13 174
DS497894.1-5 196
GG694367-8 196
TCsat13cl33 196
GG694187-15 174
GG694367-3 174
GG694367-7 174
TCsat13cl37 173
TCsat13cl43 173
TCsat13cl48 174
TCsat13cl50 174
TCsat13cl34 174
TCsat13cl21a 174
DS497837.1-11 174
TCsat13cl24b 174
TCsat13cl55 174
TCsat13cl35 174
GG694187-1 171
DS497837.1-9 174
TCsat13cl29 174
TCsat13cl42 174
TCsat13cl4a 174
TCsat13cl5b 174
GG694187-8 174
GG694187-3 174
GG694187-4 174
GG694187-9 174
GG694367-2 174
GG694367-6 174
DS497837.1-5 174
TCsat13cl49 174
GG694187-12 173
TCsat13cl4b 174
TCsat13cl13 174
DS497837.1-2 174
DS497837.1-7 174
TCsat13cl47 174
TCsat13cl21b 173
TCsat13cl24a 174
DS497837.1-8 174
DS497894.1-6 174
TCsat13cl41 174
GG694187-2 174
GG694187-7 174
DS497837.1-4 174
TCsat13cl38 174
TCsat13cl39 174
DS497837.1-12 174
DS497837.1-10 174
TCsat13cl26 173
TCsat13cl31 173
TCsat13cl52 173
TCsat13cl18b 174
TCsat13cl18a 174
TCsat13cl5a 174
TCsat13cl54 174
TCsat13cl32 174
DS497894.1-3 174
GG694367-4 174
DS497837.1-6 174
TCsat13cl40 174
TCsat13cl44 174
GG694367-9 174
DS497837.1-1 174
GG694187-5 174
GG694187-10 174
DS497837.1-3 174
GG694187-14 174
TCsat13cl27 173

```
GG694187-17     ...A..............T....C...T..TG..........C.T........................----------------------......... 174
DS497894.1-2    ..................TC..TCA.....TG..A.......C.TGC.....T..C....A.......A----------------------......... 174
GG694187-16     ..................TGG..........G..........C.T.C......................----------------------......... 174
DS497894.1-4    ..................TG..T........G..........C.T...............T........----------------------......T.. 174
GG694367-5      ..................TG..T........G..........C.T...............T........----------------------......T.. 174
GG694367-1      ..................TG..T.......TG..........C.T...............T........----------------------......... 174

                        210       220       230       240       250       260       270       280       290       300
                ....|....|....|....|....|....|....|....|....|....|....|....|....|....|....|....|....|....|....|....|
TCsat13con      GCAATCAAATTTGACATATTCAAATAAAGGGGGTAGTGGGGAATTAA-------AATGTCAAAATTCAAAAAAATT--AAAAATCAAC-CAAATTAATAT 264

DS497894.1-1    AT.....G.G............G..T.G..A..........G..CG.TCGGATCG.....GTTT..TGC.C.------G.GGCA.TC.-TTT..CG...- 266
GG694187-21     CT.....G.G...............T..............A......-------............T-----TT.C--.T..G.TGTTAT.....TT.G. 260
DS497837.1-13   ...............................................-------......................--......T...-........... 264
GG694367-10     ...............................................-------......................--......T...-........... 264
GG694187-19     ..........................GG................A..-------...................T..--T......G..-..C.A...... 264
GG694187-20     C......G.G.........C...T.T.-....T.TC....A......-------...A...............T..--T.........-........... 263
GG694187-18     ......T....................G...........A.......-------.....T......A.....T...--.......G..-........... 264
GG694187-6      ......T....................G...................-------............A......T..--.......G..-........... 264
GG694187-11     ......T....................G...................-------......................--..........-........... 264
GG694187-13     ..................................G..........T.-------......................--......T...-........... 264
DS497894.1-5    ..................C............................-------......................--......T...-........... 286
GG694367-8      ..................C....................A.......-------......................--.........G-........... 286
TCsat13cl33     ..................C....................A.......-------......................--.........G-........... 286
GG694187-15     ...........................G..........T.A......-------...................T..--..........-.....C..... 264
GG694367-3      ...............................................-------.....................A--..........-........... 264
GG694367-7      ........G......................................-------.....................A--..........-........... 264
TCsat13cl37     ...............................................-------......................--......T...-........... 263
TCsat13cl43     ...............................................-------......................--......T...-........... 263
TCsat13cl48     ...............................................-------......................--..........-........... 264
TCsat13cl50     ...............................................-------......................--..........-........... 264
TCsat13cl34     ...............................................-------......................--..........-........... 264
TCsat13cl21a    ...............................................-------......................--..........-........... 264
DS497837.1-11   ...............................................-------......................--..........-........... 264
TCsat13cl24b    ...............................................-------......................--..........-........... 264
TCsat13cl55     ...............................................-------......................--..........-........... 264
TCsat13cl35     ...............................................-------.....................A--..........-........... 264
GG694187-1      ...............................................-------............T.........--..........-........... 261
DS497837.1-9    ...............................................-------......................--......T...-........... 264
TCsat13cl29     ...............................................-------......................--......T...-........... 264
TCsat13cl42     ...............................................-------......................--......T...-........... 264
TCsat13cl4a     ...............................................-------............T.........--..........-........... 264
TCsat13cl5b     ...............................................-------...................C..--..........-........... 264
GG694187-8      ..................C............................-------......................--......T...-..T........ 264
GG694187-3      ..................C....................A.......-------......................--......T...-..T........ 264
GG694187-4      ........G.........C............................-------............A......T..--.......G..-........... 264
GG694187-9      ..................C....................A.......-------............T.........-T.......G..-........... 265
GG694367-2      ..................C............................-------......................--......T...-........... 264
GG694367-6      ..................C............................-------......................--......T...-........... 264
DS497837.1-5    ...............................................-------............T.........--......TT..-........... 264
TCsat13cl49     .............................................T.-------T.....................--......T...-........... 264
GG694187-12     ..................-............................-------......................AA....A.....-........... 264
TCsat13cl4b     ........................................-......-------......................--.........A-........... 263
TCsat13cl13     ...............................................-------............T.........--..........-........... 264
DS497837.1-2    ....................T..........................-------......................--..........-.........G. 264
DS497837.1-7    ..................C....................A.......-------......................--.........G-........... 264
TCsat13cl47     ...............................................-------......................--..........-........... 264
TCsat13cl21b    ...............................................-------......................--..........-........... 263
TCsat13cl24a    ...............................................-------..........A...........--..........-........... 264
DS497837.1-8    ...............................................-------......................--..........-........... 264
DS497894.1-6    ...............................................-------..........A...........--..........-........... 264
TCsat13cl41     ...............................................-------......................--.........A-........... 264
GG694187-2      ...............................................-------......................--.........A-........... 264
GG694187-7      ...............................................-------......................--......T...-........... 264
DS497837.1-4    ...............................................-------.............-........--......T...-........... 263
TCsat13cl38     ...............................................-------.....................A--..........-........... 264
TCsat13cl39     ...............................................-------.....................A--..........-........... 264
DS497837.1-12   ...............................................-------......................--..........-........... 264
DS497837.1-10   ...............................................-------.....................A--..........-........... 264
TCsat13cl26     ...............................................-------.....................A--..........-........... 263
TCsat13cl31     ...............................................-------.....................A--..........-........... 263
TCsat13cl52     ...............................................-------.....................A--..........-........... 263
TCsat13cl18b    ...............................................-------......................--.........A-........... 264
TCsat13cl18a    ...............................................-------......................--......T...-........... 264
TCsat13cl5a     ...............................................-------......................--..........-........... 264
TCsat13cl54     ...............................................-------......................--..........-........... 264
TCsat13cl32     ...............................................-------......................--..........-........... 264
DS497894.1-3    ..................C............................-------......................--..........-........... 264
GG694367-4      ..................C............................-------......................--.........G-........... 264
DS497837.1-6    ...............................................-------......................--..........-........... 264
TCsat13cl40     ...............................................-------......................--......T...-..T........ 264
TCsat13cl44     ...............................................-------......................--......T...-..T........ 264
GG694367-9      ...............................................-------..........A...........--..........-........... 264
DS497837.1-1    TT.....G.G...............T.G.......C....A......-------...................T..--......T...-..T........ 264
GG694187-5      ...........................G...................-------......................--......T...-........... 264
GG694187-10     ...........................G...................-------......................--......T...-........... 264
DS497837.1-3    T......G.G.....................................-------......................--T.........-........... 264
```

```
GG694187-14     T......G.G.....................................-------......................--..........-........... 264
TCsat13cl27     T......G.G.....................................-------......................--..........-........... 263
GG694187-17     TT.....G.G...............TTG............A......-------......................--..........-........... 264
DS497894.1-2    C......T.G...............T.G............A......-------...................T..--.......C..-.T......... 264
GG694187-16     TTG...CG.G...............T.G............A......-------...A............T..T..--......T...-..T........ 264
DS497894.1-4    TT.....G.G..........T....T.G............A......-------............T.........--..........-........... 264
GG694367-5      TT.....G.G..........T....T.G............A......-------............T.........--..........-........... 264
GG694367-1      TT.....G.G..........T....T.G............A......-------............T......T..--..........-........... 264


                ....|.
TCsat13con      AT     266

DS497894.1-1    ------ 266
GG694187-21     ..GTTT 266
DS497837.1-13   ..---- 266
GG694367-10     ..---- 266
GG694187-19     ..---- 266
GG694187-20     ..---- 265
GG694187-18     ..---- 266
GG694187-6      ..---- 266
GG694187-11     ..---- 266
GG694187-13     ..---- 266
DS497894.1-5    ..---- 288
GG694367-8      ..---- 288
TCsat13cl33     ..---- 288
GG694187-15     ..---- 266
GG694367-3      ..---- 266
GG694367-7      ..---- 266
TCsat13cl37     ..---- 265
TCsat13cl43     ..---- 265
TCsat13cl48     ..---- 266
TCsat13cl50     ..---- 266
TCsat13cl34     ..---- 266
TCsat13cl21a    ..---- 266
DS497837.1-11   ..---- 266
TCsat13cl24b    ..---- 266
TCsat13cl55     ..---- 266
TCsat13cl35     ..---- 266
GG694187-1      ..---- 263
DS497837.1-9    ..---- 266
TCsat13cl29     ..---- 266
TCsat13cl42     ..---- 266
TCsat13cl4a     ..---- 266
TCsat13cl5b     ..---- 266
GG694187-8      ..---- 266
GG694187-3      ..---- 266
GG694187-4      ..---- 266
GG694187-9      ..---- 267
GG694367-2      ..---- 266
GG694367-6      ..---- 266
DS497837.1-5    ..---- 266
TCsat13cl49     ..---- 266
GG694187-12     ..---- 266
TCsat13cl4b     ..---- 265
TCsat13cl13     ..---- 266
DS497837.1-2    ..---- 266
DS497837.1-7    ..---- 266
TCsat13cl47     ..---- 266
TCsat13cl21b    ..---- 265
TCsat13cl24a    ..---- 266
DS497837.1-8    ..---- 266
DS497894.1-6    ..---- 266
TCsat13cl41     ..---- 266
GG694187-2      ..---- 266
GG694187-7      ..---- 266
DS497837.1-4    ..---- 265
TCsat13cl38     ..---- 266
TCsat13cl39     ..---- 266
DS497837.1-12   ..---- 266
DS497837.1-10   ..---- 266
TCsat13cl26     ..---- 265
TCsat13cl31     ..---- 265
TCsat13cl52     ..---- 265
TCsat13cl18b    ..---- 266
TCsat13cl18a    ..---- 266
TCsat13cl5a     ..---- 266
TCsat13cl54     ..---- 266
TCsat13cl32     ..---- 266
DS497894.1-3    ..---- 266
GG694367-4      ..---- 266
DS497837.1-6    ..---- 266
TCsat13cl40     ..---- 266
TCsat13cl44     ..---- 266
GG694367-9      ..---- 266
DS497837.1-1    ..---- 266
GG694187-5      ..---- 266
GG694187-10     ..---- 266
```

```
DS497837.1-3     ..---- 266
GG694187-14      ..---- 266
TCsat13cl27      ..---- 265
GG694187-17      ..---- 266
DS497894.1-2     ..---- 266
GG694187-16      ..---- 266
DS497894.1-4     ..---- 266
GG694367-5       ..---- 266
GG694367-1       ..---- 266
```

C) TCsat14

```
                           10        20        30        40        50        60        70        80        90       100
                 ....|....|....|....|....|....|....|....|....|....|....|....|....|....|....|....|....|....|....|....|
TCsat14con       TTACAAAACGCAATAAAGCAGAAGTGAGTATATGTTGACACCAAGTCTTTTTTGTTTTATGAAGTGTATTTTAGCCTGTAAGCGTTGAAAATGTCGAGGA 100
                                                  GTYRAC
                                                  HincII
ChLG3-1          .....................................C.............................................................. 100
DS497883.1-1     A....................................C.............................................................. 100
TCsat14cl24b     .....................................C..........................................G......G............ 100
ChLG7-16         .....................................C.............................................................. 100
TCsat14cl19b     .................................................................................................... 100
TCsat14cl14b     ..........................................................................................G......... 100
TCsat14cl30a     ...............................G.................................................................... 100
TCsat14cl31      .....................................C.............................................................. 100
ChLG7-15         .....................................C.............................................................. 100
TCsat14cl9b      .....................................C.............................................................. 100
TCsat14cl5b      .....................................C.............................................................. 100
TCsat14cl23a     .......................................................................................G............ 100
ChLG7-14         .......................................................................................G............ 100
TCsat14cl30b     .......................................................................................G............ 100
TCsat14cl29b     ...............................................-.................................................... 99
TCsat14cl32      .................................................................................................... 100
ChLG7-26         .....................................C.............................................................. 100
TCsat14cl25a     .....................................C.............................................................. 100
TCsat14cl3b      ................................................................................G................... 100
TCsat14cl17      .................................................................................................... 100
ChLG7-12         ................................................................................-................... 99
TCsat14cl25b     .................................................................................................... 100
TCsat14cl7       .....................................C.............................................................. 100
TCsat14cl10      .....................................C...................................A.......................... 100
TCsat14cl28b     .................................................................................................... 100
TCsat14cl33      .....................................C.............................................................. 100
TCsat14cl11b     .....................................C.......................................................C...... 100
TCsat14cl19a     .................................................................................................... 100
TCsat14cl22a     ...................G................................................................................ 100
ChLG7-2          .................................................................................................... 100
ChLG7-34         .................................................................................................... 100
TCsat14cl12b     .................................................................................................... 100
ChLG7-27         .................................................................................................... 100
TCsat14cl18a     .................................................................................................... 100
ChLG7-11         .....................................C.............................................................. 100
TCsat14cl23b     .....................................C.............................................................. 100
ChLG7-1          .....................................C.............................................................. 100
ChLG7-18         .....................................C.............................................................. 100
ChLG7-28         .....................................C.............................................................. 100
TCsat14cl9a      .....................................C.............................................................. 100
TCsat14cl14a     .....................................C.............................................................. 100
ChLG7-4          .....................................C.............................................................. 100
ChLG7-8          .....................................C.............................................................. 100
ChLG7-21         .....................................C.............................................................. 100
ChLG7-23         .....................................C.............................................................. 100
ChLG7-31         .....................................C.............................................................. 100
ChLG7-35         .....................................C.............................................................. 100
TCsat14cl1a      .....................................C.............................................................. 100
TCsat14cl6       .....................................C.............................................................. 100
TCsat14cl16a     .....................................C.............................................................. 100
TCsat14cl18b     .....................................C.............................................................. 100
TCsat14cl22b     .....................................C.............................................................. 100
ChLG7-5          .....................................C.............................................................. 100
TCsat14cl2       .....................................C.............................................................. 100
TCsat14cl3a      .....................................C.............................................................. 100
TCsat14cl21      .....................................C.............................................................. 100
ChLG7-6          .....................................C.............................................................. 100
TCsat14cl16b     .....................................C.............................................................. 100
TCsat14cl5a      .................................................................................................... 100
ChLG7-17         ...............................................-.................................................... 99
ChLG7-3          .................................................................................................... 100
ChLG7-7          .................................................................................................... 100
ChLG7-13         .................................................................................................... 100
ChLG7-29         .................................................................................................... 100
ChLG7-30         .................................................................................................... 100
```

```
ChLG7-32        .................................................................................................... 100
TCsat14cl1b     .................................................................................................... 100
TCsat14cl11a    .................................................................................................... 100
TCsat14cl24a    .................................................................................................... 100
TCsat14cl26     .................................................................................................... 100
TCsat14cl35     .................................................................................................... 100
ChLG7-9         .................................................................................................... 100
ChLG7-22        .................................................................................................... 100
ChLG7-33        .................................................................................................... 100
ChLG7-10        .................................................................................................... 100
ChLG7-20        .................................................................................................... 100
ChLG7-24        .................................................................................................... 100
ChLG7-25        .................................................................................................... 100
TCsat14cl12a    .................................................................................................... 100
TCsat14cl29a    .................................................................................................... 100
TCsat14cl34     .................................................................................................... 100
ChLG7-19        .................................................................................................... 100
TCsat14cl28a    .................................................................................................... 100

                         110       120       130       140       150       160       170       180       190       200
                ....|....|....|....|....|....|....|....|....|....|....|....|....|....|....|....|....|....|....|....|
TCsat14con      AACTGTTGCACCTAAAGCATGGGAAAACAAGGTATTTATATTTTACTTGAAGTGAATTTTTGTGATAATTTCCAC--AAAAAAAAGTGTGGTTCAGTTCT 198

ChLG3-1         ...................A..A....................................................--....................... 198
DS497883.1-1    ......................A....................................................-A....................... 199
TCsat14cl24b    ...........................................................................--....................... 198
ChLG7-16        ...........................................................................---...................... 197
TCsat14cl19b    ...........................................................................---..G................... 197
TCsat14cl14b    ........................................C..........A.......................--....................... 198
TCsat14cl30a    ...........................................................................-A....................... 199
TCsat14cl31     ...................................................A.......................--....................... 198
ChLG7-15        ...................................................A.......................--....................... 198
TCsat14cl9b     ...........................................................................---...................... 197
TCsat14cl5b     ...........................................................................---...................... 197
TCsat14cl23a    ...........................................................................---...................... 197
ChLG7-14        ...........................................................................---...................... 197
TCsat14cl30b    ...........................................................................---...................... 197
TCsat14cl29b    ...........................................................................-A.............C......... 198
TCsat14cl32     ...........................................................................-A.............C......... 199
ChLG7-26        ...........................................................................-A.............C......... 199
TCsat14cl25a    ...........................................................................-A.............C......... 199
TCsat14cl3b     ...........................................................................---...................... 197
TCsat14cl17     .........................................................-.................---...................... 196
ChLG7-12        .........C.................................................................---...................... 196
TCsat14cl25b    ...........................................................................---...................... 197
TCsat14cl7      ...........................................................................---...........C.......... 197
TCsat14cl10     ...........................................................................---...................... 197
TCsat14cl28b    ...........................................................................---...................... 197
TCsat14cl33     ...........................................................................---.............C........ 197
TCsat14cl11b    ...........................................................................--....................... 198
TCsat14cl19a    ...........................................................................-A....................... 199
TCsat14cl22a    ...........................................................................--....................... 198
ChLG7-2         ...................................................A.......................-A....................... 199
ChLG7-34        ...................................................A.......................--....................... 198
TCsat14cl12b    ...................................................A.......................--....................... 198
ChLG7-27        ...........................................................................-A.......C............... 199
TCsat14cl18a    ...........................................................................-A.......C............... 199
ChLG7-11        ...........................................................................-A....................... 199
TCsat14cl23b    ...........................................................................-A...........C........... 199
ChLG7-1         ...........................................................................-A....................... 199
ChLG7-18        ...........................................................................-A....................... 199
ChLG7-28        ...........................................................................-A....................... 199
TCsat14cl9a     ...........................................................................-A....................... 199
TCsat14cl14a    ...........................................................................-A....................... 199
ChLG7-4         ...........................................................................---...................... 197
ChLG7-8         ...........................................................................---...................... 197
ChLG7-21        ...........................................................................---...................... 197
ChLG7-23        ...........................................................................---...................... 197
ChLG7-31        ...........................................................................---...................... 197
ChLG7-35        ...........................................................................---...................... 197
TCsat14cl1a     ...........................................................................---...................... 197
TCsat14cl6      ...........................................................................---...................... 197
TCsat14cl16a    ...........................................................................---...................... 197
TCsat14cl18b    ...........................................................................---...................... 197
TCsat14cl22b    ...........................................................................---...................... 197
ChLG7-5         ...........................................................................--....................... 198
TCsat14cl2      ...........................................................................--....................... 198
TCsat14cl3a     ...........................................................................--....................... 198
TCsat14cl21     ...........................................................................--....................... 198
ChLG7-6         ...........................................................................----..................... 196
TCsat14cl16b    ...........................................................................AA....................... 200
TCsat14cl5a     .........................................................C.................AA....................... 200
ChLG7-17        ...........................................................................-A....................... 199
ChLG7-3         ...........................................................................--....................... 198
ChLG7-7         ...........................................................................--....................... 198
ChLG7-13        ...........................................................................--....................... 198
```

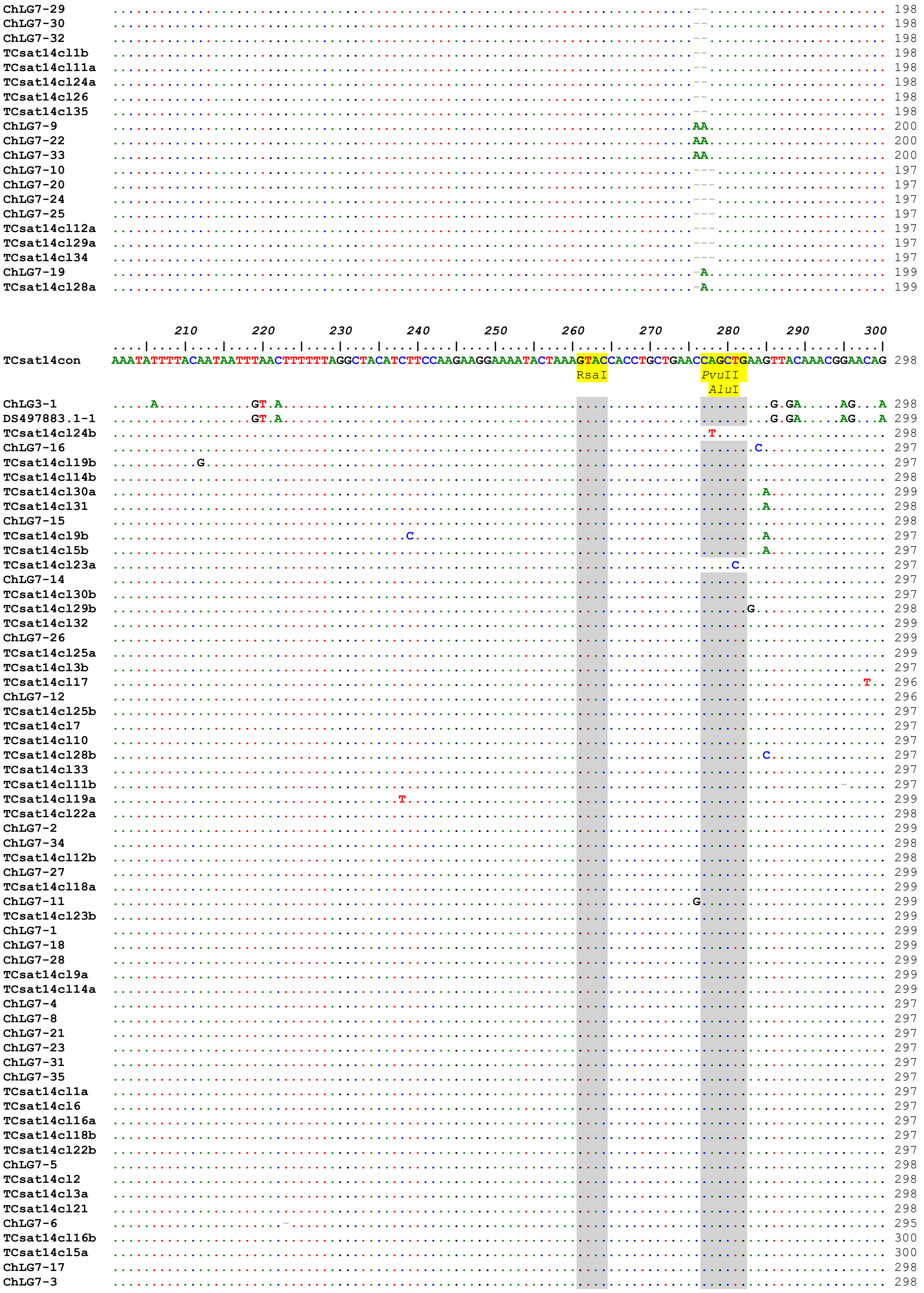
TCsat14con
AAATATTTTACAATAATTTAACTTTTTTAGGCTACATCTTCCAAGAAGGAAAATACTAAAGTACCACCTGCTGAACCAGCTGAAGTTACAAACGGAACAG 298
RsaI
PvuII
AluI
210 220 230 240 250 260 270 280 290 300

```
ChLG7-7          .................................................................................................... 298
ChLG7-13         .................................................................................................... 298
ChLG7-29         .................................................................................................... 298
ChLG7-30         .................................................................................................... 298
ChLG7-32         .................................................................................................... 298
TCsat14cl1b      .................................................................................................... 298
TCsat14cl11a     .................................................................................................... 298
TCsat14cl24a     .................................................................................................... 298
TCsat14cl26      .................................................................................................... 298
TCsat14cl35      .................................................................................................... 298
ChLG7-9          .................................................................................................... 300
ChLG7-22         .................................................................................................... 300
ChLG7-33         .................................................................................................... 300
ChLG7-10         .................................................................................................... 297
ChLG7-20         .................................................................................................... 297
ChLG7-24         .................................................................................................... 297
ChLG7-25         .................................................................................................... 297
TCsat14cl12a     .................................................................................................... 297
TCsat14cl29a     .................................................................................................... 297
TCsat14cl34      .................................................................................................... 297
ChLG7-19         .................................................................................................... 299
TCsat14cl28a     .................................................................................................... 299
```

```
                         310
                 ....|....|..
TCsat14con       ATGAAAAAAAAA 310
ChLG3-1          CC.......G.. 310
DS497883.1-1     CC.......G.. 311
TCsat14cl24b     ............ 310
ChLG7-16         ...........T 309
TCsat14cl19b     ............ 309
TCsat14cl14b     ............ 310
TCsat14cl30a     ............ 311
TCsat14cl31      ............ 310
ChLG7-15         ............ 310
TCsat14cl9b      ............ 309
TCsat14cl5b      ............ 309
TCsat14cl23a     ............ 309
ChLG7-14         ............ 309
TCsat14cl30b     ............ 309
TCsat14cl29b     ............ 310
TCsat14cl32      ............ 311
ChLG7-26         ............ 311
TCsat14cl25a     ............ 311
TCsat14cl3b      ...........- 308
TCsat14cl17      ............ 308
ChLG7-12         ............ 308
TCsat14cl25b     ...........T 309
TCsat14cl7       ............ 309
TCsat14cl10      ............ 309
TCsat14cl28b     ............ 309
TCsat14cl33      ............ 309
TCsat14cl11b     ............ 309
TCsat14cl19a     ...........- 310
TCsat14cl22a     ............ 310
ChLG7-2          ............ 311
ChLG7-34         ............ 310
TCsat14cl12b     ............ 310
ChLG7-27         ............ 311
TCsat14cl18a     ............ 311
ChLG7-11         ............ 311
TCsat14cl23b     ............ 311
ChLG7-1          ............ 311
ChLG7-18         ............ 311
ChLG7-28         ............ 311
TCsat14cl9a      ............ 311
TCsat14cl14a     ............ 311
ChLG7-4          ............ 309
ChLG7-8          ............ 309
ChLG7-21         ............ 309
ChLG7-23         ............ 309
ChLG7-31         ............ 309
ChLG7-35         ............ 309
TCsat14cl1a      ............ 309
TCsat14cl6       ............ 309
TCsat14cl16a     ............ 309
TCsat14cl18b     ............ 309
TCsat14cl22b     ............ 309
ChLG7-5          ............ 310
TCsat14cl2       ............ 310
TCsat14cl3a      ............ 310
TCsat14cl21      ............ 310
ChLG7-6          ............ 307
TCsat14cl16b     ............ 312
TCsat14cl5a      ............ 312
ChLG7-17         ............ 310
ChLG7-3          ............ 310
```

ChLG7-7	....	310
ChLG7-13	....	310
ChLG7-29	....	310
ChLG7-30	....	310
ChLG7-32	....	310
TCsat14cl1b	....	310
TCsat14cl11a	....	310
TCsat14cl24a	....	310
TCsat14cl26	....	310
TCsat14cl35	....	310
ChLG7-9	....	312
ChLG7-22	....	312
ChLG7-33	....	312
ChLG7-10	....	309
ChLG7-20	....	309
ChLG7-24	....	309
ChLG7-25	....	309
TCsat14cl12a	....	309
TCsat14cl29a	....	309
TCsat14cl34	....	309
ChLG7-19	....	311
TCsat14cl28a	....	311

Figure S6. The alignments of TCsat12 (**A**), TCsat13 (**B**), and TCsat14 (**C**) monomers. The consensus sequences representing the most prevailing nucleotide at each position are listed as the first sequences. Monomers extracted from the Tcas5.2 assembly are labeled “ChLG” (chromosome linkage groups), “DD” (unplaced scaffolds) or “GG” (unplaced singletons). PCR-generated copies are designated as “TCsat12/13/14” followed by the clone number, and the suffixes “a” and “b” denote two monomeric copies obtained from the same cloned insert. Identities between the compared monomers and the consensus sequence are indicated by dots (.), and dashes (-) indicate alignment gaps. The yellow boxes in the consensus sequences mark recognition sites for specific restriction endonucleases, and the corresponding positions in the monomers are indicated by gray boxes.

.
