# Supplementary material for: The Low-Copy-Number Satellite DNAs of the Model Beetle Tribolium castaneum
Source: Genes (Basel). 2023 Apr 28;14(5):999. doi: 10.3390/genes14050999 (PMC10218199; doi:10.3390/genes14050999)
Supplement: Supplementary file 1 [file genes-14-00999-s001.zip › Supplementary materials/Figure S7.pdf]

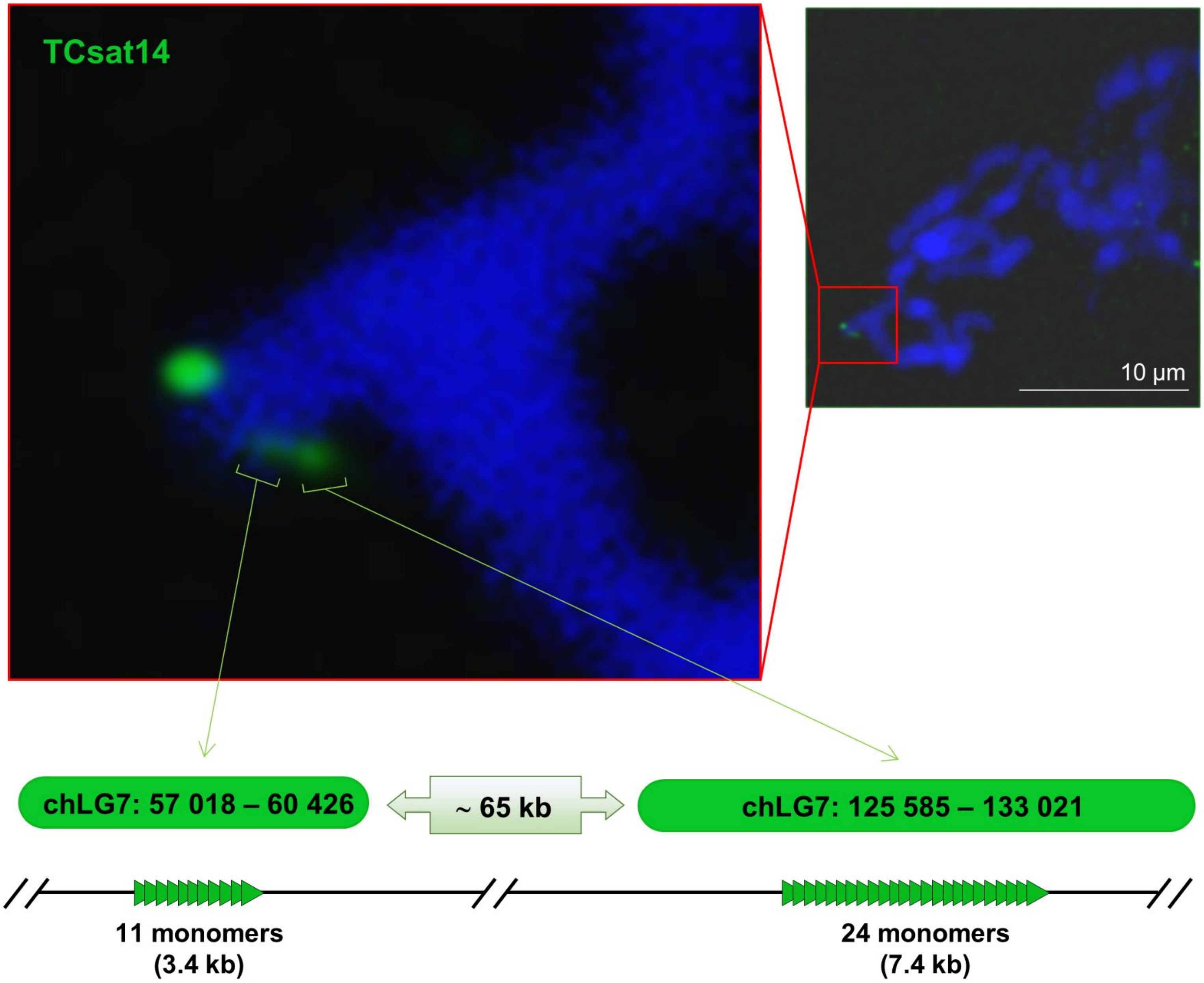

Figure S7. Physical localization of the TCsat14 satDNA on *T. castaneum* chromosomes revealed by fluorescence *in situ* hybridization. Chromosomes were stained with 4',6-diamidino-2-phenylindole (blue fluorescence) and hybridized with TCsat14 satDNA probe visualized by fluorescein isothiocyanate (green fluorescence). At the enlarged panel, it can be seen on one of the chromatids that the signal consists of two very closely spaced signals (one weaker and one stronger) that correspond to the position of two TCsat14 satDNA arrays (3.4 kb and 7.4 kb long, respectively) 65 kb apart, at the beginning of chromosome chLG7, according to the Tcas5.2 genome assembly. The scheme illustrates the position of the two TCsat14 arrays with the exact coordinates in the genome assembly Tcas5.2.
