# Supplementary material for: The Low-Copy-Number Satellite DNAs of the Model Beetle Tribolium castaneum
Source: Genes (Basel). 2023 Apr 28;14(5):999. doi: 10.3390/genes14050999 (PMC10218199; doi:10.3390/genes14050999)
Supplement: Supplementary file 1 [file genes-14-00999-s001.zip › Supplementary materials/Table S1.pdf]

Table S1. The output of seven clustering analyses (T1-T7) performed by the TAREAN pipeline using Illumina paired-end whole genome sequence reads of the red flour beetle *T. castaneum*. The suffixes “H” and “L” in the cluster numbers indicate the status of satellite repeats identified as “**H**igh putative” and “**L**ow putative” by the TAREAN analysis, respectively.

**TAREAN clustering analysis**		**T1**		**T2**		**T3**		**T4**		**T5**		**T6**		**T7***	
No. of analysed reads		230,000		575,000		1,150,000		1,500,000		1,720,000		2,300,000		1,500,000 (**1,197,688)	
Genome coverage		0.1x		0.25x		0.5x		0.65x		0.75x		1x		0.65x	
No. of automatically filtered abundand reads		35,542 (15.45%)		88,012 (15.31%)		176,984 (15.39%)		307,506 (20.5%)		349,298 (20.31%)		353,592 (15.37%)		30,2312*** (20.15%)	
Proportion of reads in top clusters		20%		25%		27%		24%		24%		31%		24%	
No. of reads in clusters		105,545 (45.89%)		308,282 (53.61%)		719,077 (62.53%)		1,013,181 (67.55%)		1,186,942 (69.00%)		1,708,455 (74.28%)		1,019,277 (69.63%)	
No. of clusters		8,601		32,304		93,308		139,090		167,962		248,696		145,696	
No. of singlets		124,455		266,718		430,923		486,819		533,058		591,545		455,613	
No. of satellites		12		19		26		49		47		72		37	
No. of high putative satellites		2		3		3		7		7		7		16	
No. of low putative satellites		10		16		23		42		40		65		21	
Satellite DNA	Average genome content %	Cluster	Genome content (%)	Cluster	Genome content (%)	Cluster	Genome content (%)	Cluster	Genome content (%)	Cluster	Genome content (%)	Cluster	Genome content (%)	Cluster	Genome content (%)
TCsat12	0.0461	54H	0.0490	64L	0.0510	71L	0.0480	88L	0.0470	82L	0.0540	84L	0.0500	121H	0.0240
TCsat13	0.0184	102L	0.0170	104L	0.0180	110L	0.0190	136L	0.0180	148L	0.0160	124L	0.0230	143H	0.0180
TCsat14	0.0154	95L	0.0190	137L	0.0120	129H	0.0150	157L	0.0120	152L	0.0160	148L	0.0130	131L	0.0210
TCsat15	1.6857	4L	1.6000	4L	1.7000	4L	1.7000	2L	1.7000	2L	1.7000	3L	1.8000	2L	1.6000
TCsat16	0.0059	-		239H	0.0061	282H	0.0044	231H	0.0060	273H	0.0041	185H	0.0057	204H	0.0088
TCsat17	0.0077	-		168L	0.0092	244L	0.0055	223L	0.0065	191L	0.0091	165L	0.0084	226H	0.0073
TCsat18	0.0892	-		48L	0.0800	48L	0.0960	55L	0.0950	55L	0.0920	-		210H	0.0830
TCsat19	0.0174	-		-		123L	0.0160	142L	0.0160	136L	0.0180	142L	0.0160	129L	0.0210
TCsat20	0.0045	-		-		286L	0.0043	275H	0.0042	299L	0.0035	224L	0.0041	251H	0.0063
TCsat21	0.0041	-		-		276L	0.0045	253L		291L	0.0037	225L	0.0041	343H	0.0040
TCsat22	0.0040	-		-		-		367H	0.0029	326L	0.0032	245L	0.0036	258L	0.0061
TCsat23	0.0078	153L	0.0100	277L	0.0052	197L	0.0078	196L	0.0081	-		-		-	
TCsat24	0.0898	-		56L	0.0660	-		52L	0.0980	52L	0.0980	58L	0.0970	-	
TCsat25	0.0027	-		-		405L	0.0025	337L	0.0032	410L	0.0023	291H	0.0027	-	
TCsat26	0.0030	-		-		340L	0.0033	-		371L	0.0026	318L	0.0024	360H	0.0038

TCsat27	0.0040	-	-	-		-		341H	0.0030	254L	0.0033	270H	0.0058
TCsat28	0.0036	-	-	-		297L	0.0038	307H	0.0034	239H	0.0037	-	
TCsat29	0.0028	-	-	-		-		275L	0.0041	314L	0.0025	629L	0.0018
TCsat30	0.0019	-	-	-		447L	0.0022	532L	0.0016	404L	0.0020	-	
TCsat31	0.0018	-	-	-		543L	0.0017	433L	0.0021	477L	0.0017	-	
TCsat32	0.0033	-	-	-		344L	0.0031	-		277L	0.0030	365L	0.0037
TCsat33	0.0017	-	-	564L	0.0017	625H	0.0015	-		418L	0.0019	-	
TCsat34	0.0020	-	-	-		-		561L	0.0016	472L	0.0017	465H	0.0026
TCsat35	0.0018	-	-	-		466L	0.0021	557H	0.0016	497L	0.0017	-	
TCsat36	0.0020	-	-	425L	0.0023	518L	0.0018	-		447L	0.0018	-	
TCsat37	0.0470	-	-	-		CL87L	0.0470	87L	0.0470	-		-	
TCsat38	0.0070	-	-	153L	0.0110	-		-		-		430L	0.0029
TCsat39	0.0018	-	-	-		428L	0.0023	-		598L	0.0013	-	
TCsat40	0.0020	-	-	-		570L	0.0017	-		-		514H	0.0023
TCsat41	0.0016	-	-	-		505L	0.0019	-		636L	0.0013	-	
TCsat42	0.0016	-	-	-		-	-	602L	0.0014	-		637L	0.0018
TCsat43	0.0016	-	-	-		647L	0.0015	-		487L	0.0017	-	
TCsat44	0.0021	-	-	-		-		-		408L	0.0019	522H	0.0023
TCsat45	0.0017	-	-	-		-		-		747L	0.0011	509H	0.0023
TCsat46	0.0017	-	-	-		452L	0.0021	-		614L	0.0013	-	
TCsat47	0.0019	-	-	-		596L	0.0016	430L	0.0022	-		-	
TCsat48	0.0017	-	-	-		467L	0.0021	-		641L	0.0013	-	
TCsat49	0.0017	-	-	-		500L	0.0019	-		561L	0.0014	-	
TCsat50	0.0024	-	-	-		419L	0.0024	-		-		-	
TCsat51	0.0043	-	-	-		271L	0.0043	-		-		-	
TCsat52	0.0013	-	-	-		686L	0.0013	-		-		-	
TCsat53	0.0017	-	-	-		540L	0.0017	-		-		-	
TCsat54	0.0022	-	-	-		-		-		-		541L	0.0022
TCsat55	0.0023	-	-	-		-		-		-		524L	0.0023
TCsat56	0.0024	-	-	-		-		-		-		499L	0.0024
TCsat57	0.0013	-	-	-		698L	0.0013	-		-		-	

* For TAREAN clustering analysis T7, an initial subset of 1,500,000 randomly subsampled Illumina reads was selected.

** Using Geneious Prime software, the reads belonging to the major satDNA TCAST were excluded from the initial subset of 1,500,000 reads, and the remaining 1,197,688 TCAST-free reads were subjected to the TAREAN analysis.

*** In the initial subset of 1,500,000 Illumina reads, 302,312 reads were recognized as the reads belonging to the major satDNA TCAST by Geneious Mapper tool. The TCAST-related reads were excluded from the subsequent clustering analysis.
