# Supplementary material for: The Low-Copy-Number Satellite DNAs of the Model Beetle Tribolium castaneum
Source: Genes (Basel). 2023 Apr 28;14(5):999. doi: 10.3390/genes14050999 (PMC10218199; doi:10.3390/genes14050999)
Supplement: Supplementary file 1 [file genes-14-00999-s001.zip › Supplementary materials/Table S2.pdf]

Table S2. Consensus sequences and GenBank accession numbers of the 46 *T. castaneum* satDNAs identified in this work.

SatDNA	Consensus sequence	GenBank Accession Number
**TCsat12**	TCAAAAATTATAAACAATACAACTTTCCAACCCCTTCCCAGGGCTCGATTTTTTTTAAACTTTTTTATAATGTAGAGTATTAAATGCTCTTT<br>CGACCTGCCGTAACAAGAAAGTGGCACATTGTCGATAACACATAGAAAAAAAATAAAAACCA	OQ551416
**TCsat13**	GTTATAGTAGGGAAGCTCAGGATCAATTTAAAATTATTTTCAGAATTTTTTCTCATTTCCTACCCCAAAACTTATAGGGGTAAATTTGGCC<br>AAAAATTTGAAAATCTTTATTTACATTCGGATCGAGTTCGTTTTTTGTACATGGGTACCCTCTATGGTCCTGATTATGCTCCAGCAATCAA<br>ATTTGACATATTCAAATAAAGGGGGTAGTGGGGAATTAAAATGTCAAAATTCAAAAAAATTAAAAATCAACCAAATTAATATAT	OQ551417
**TCsat14**	TTACAAAACGCAATAAAGCAGAAGTGAGTATATGTTGACACCAAGTCTTTTTTGTTTTATGAAGTGTATTTTAGCCTGTAAGCGTTGAAAA<br>TGTCGAGGAAACTGTTGCACCTAAAGCATGGGAAAACAAGGTATTTATATTTTACTTGAAGTGAATTTTTGTGATAATTTCCACAAAAAAA<br>GTGTGGTTCAGTTCTAAATATTTTACAATAATTTAACTTTTTTAGGCTACATCTTCCAAGAAGGAAAATACTAAAGTACCACCTGCTGAAC<br>CAGCTGAAGTTACAAACGGAACAGATGAAAAAAAAA	OQ551418
**TCsat15**	AGGAAATTTTAGCAAAAAAATTGAAAATTTTAAATCTCGTGAAAAGTTGGTATAATACAGAAAATGAGCCGCTGAATTCAATGGAACAAAC<br>CGCATTGCTCTACGACTTTTAGTTTTTTTTTTATGAATTTTTTTAGTGAAAAACTTTGATCGCCTCTGTAGCCGGAACCGTTGCCCGGAGC<br>GGCTCCGGGTTTGGTCGAAAAAATAGATAAAATTAAGCGCTATCAGATGGTTTTTTGTTCGTTGCTCTAAGTCTTACAGTTTCCGAGAAA<br>AACGCAAAAAAAGGTTTCCATTTTCACTTTTTTTCAATTTTCAACCGCCAATTACGGCGAAACTATTAGAGTTATCGAGAAACGGAAAAAT<br>GGAGGACAACCGGAATAAAAAACTCTACAAAATGGCATAGGACTCAAGGCGATATCTCGCAAAAAATTTTTCAATTTTCAAACGCCGATT<br>ACGGCCAAACTAAAAGAGCTAGGAGAAAACGCTTTTTTAGATCGGAAAGAGCACATCAAAATCTATAAGATAGAATCGGTTTTATTTGAT<br>TTTGAGACACTTTAAAAATTGACCGATTTTAAGGGGGGGGTGTTAACTTTTTTTTAATTTTTTTTTATTTTCTCATTTACGGCTAAACTATTC<br>GAAAAAGTGGAACTGTTTTGATCCATACCTATGCAAAATGATCCGGGGAATCGATTGGCATCGAGAAAATTGCCAAATTCCCAATAGTTT<br>TTTAGTTATGAATTTTTTTAAAATTTTACCAATTTTTGCATTTATCTGCAATTTGCTCGAAAACTATTAGTCGGAGAACAATAATTTTTTTTGA<br>AAAAAATAGATAATTTAAAGAGCTTTCCAACGATAATACACTTCATGGGGTTATCTCTGATAGTTCCGGAGCTATTGCTCGACAAATGTT<br>CCGGGTACCCAAAATCGACAAAAACTGCGACGAAAATTGAAAAAATCAAACATCAAAAAATCGAACATATGGACTTTTTGTGGACTTTTA<br>ACAACTTTTGTGGGTTGTTAAGACATGTAGAAGTCCATAAAAATTTACTGGAGTGAGTTTAAAAAAAAAATTTTTTTTCACCTCTTTGAAGTT<br>AAGTGTATTATTACTC	OQ551419
**TCsat16**	AAAATGGCAGCGGTCCAGCGGTTAATGTGCGTCAGAGTTAGGAAGACATTACGCAGTGTGACAATAATTGTACAGTAAATTCCTAATTT<br>AGGAATAATTTAGGGATTTCACGTTGCATCGAAACCTTTCGGAACATGATAGCTTTAGTGTACAGGGTGCCGCATTTTGGATATCCGAAT<br>AGGAGGGAATCTAATAATATAGGGCAAGATGCAATAAAAAATTGTACTACTGCTAAATCGAATCCAACTTGTTCAATTAACAGTAAATATA<br>TGTATCTTGTCACAATCTTAGTTTTTGATATAGTTGATGTCATTAGCAATCTTAAATCAA	OQ551420
**TCsat17**	GACAAGGCTCCTTCTCCTAAAGACAAAACTCCTACACCCGAAAAGCCCTCTGAGATTAAACTTAAACCTGAAGAAATCACGGAAAAGGA<br>AAAATCACCTGAGAAAGTAGAACTGAAAGAG	OQ551421
**TCsat18**	TTGGCAACGTGTTTTAAACACGTGTCCGCGCCATTTTTTAAATTTAATTACCGATTTCGTGCGATTTTTTAGG	OQ551422
**TCsat19**	TACATACATAAAGTTATTTATCGAAAATTTGTATACTGATCTTGTATTTTTTTATTTTCAGTAGGTCTATTCTAAGAAGAAGAGGGAACGAA<br>GAGGACGCCCAGGGTGAGTGAATTTTAATTTTTTCTAAGTGAGGTGAAGTGTGTGAAGTGAAAATGTGAGTGCAACATTTTAGAACACA<br>GTAAGTATTTATAAGTATCAAAATATGAGTGAAAATTAATTAATTAATTGTAGATGAAACGTAATTCTCTCAAAGCTTTTGAAGAAAAATTT<br>CCAGACATTTGACGAAGCATCTCTTAAATAATGAAGAAAAACATTCACAGCAAGCTCGAGACTAAGTATATTGTACGAATTTGATTTTGTT<br>TTTTTTTCATTACTAATTTTTTCAATTTTTTGTTTCAGATGACTCGAGTGATTGAAAGTGCGTGTTGTGGATTTTCGAGTGTTTAACAGACA<br>TTTAGTAGTGCGCGATTTTTTAAATTTCGAGTGATTAAAGTGCCAATGGAAGGGATCTTCAGGGAACCTGGACGCTATAATCGTGCTAC<br>AAGGATCAGGATTAAAGGGTTAGTTGTCAAAGTTTTTTTTTCTAAAAAAATTAAGTATAAATCCGTAACATTA	OQ551423
**TCsat20**	TAATCTGGATGATGAGATGCTTCCTTTACACACACTAAACACATAATTTTAAAAATCCCGAACTCGAAAATTTTTCATGCACTTTGAATAA<br>CTTTTCTTCCCTCTTTCCTTTACTTTCTCTTCGTAATTTCTTCAAAACTCCTT	OQ551424
**TCsat21**	TGTTAATTCGTTTCGAGTCGCAATAAATTTGTTAATTTGAAAATAAAATTTAATTCGCGCTTCAAATTAATAAAAATGTACTCGGACCGCAT<br>TCACGTTCAA	OQ551425

**TCsat22**	AAAAACAAAACAGAAGAAACAATTAAATTAAGAGACTAATGGTATTCTGAATAAATAGCTTGATCATTGAAAAACTGAAAAAATTTGGAAC<br>CAAAAAGCTGAAACCCTGAGAAATCGAAAAATTTGGAACTGAAAATGTGAAAGAAACCGCGAGTGACCTACAGATTGACAAGCTTAAAA<br>TTTTAGAAGATGACAAGCTAAAAATCTGGAGGCTGAAAAGGCTAAAAAACTGAGCTAAACTAATAAACTTAACTAGTTAAAAACTAAGAA<br>ACTAAAAAAAG	OQ551426
**TCsat23**	TTTAATATTATTAATAAAATGTTGACATATTTCAAACTAAGGGTTGGATGTCTTCCCAAACTTTTAGAGGGTTGAAATTTTCAGGAAAGGT<br>AGAGTATTTAATGGGCTTTCATGTGGAATGGGTTAAATGCGTCGACAAAAAGAAATAGTACTAGAAAAAAATA	OQ551427
**TCsat24**	CAAAAAATTCAAACAATGTCAAAGATGTTTTTTAAAAATTTTAGACCTCAAAAAGAAACTCCGAAATTCTCTTAGCTTTCGTTTTTCATTGG<br>TCCAGAGCGCATGCGCATGCGCGTCTGACCTGTACATTTTCCCCACCAAACGTCCATAGATCACGGTAACTAAAGGGCGTACAATCAT<br>CAAACAACTTCTAAAGTGCTTAGGTCATGCTCCCCCGACATATGAAAAAAAAAACAGATTTTTATCTCAATTAGATCTAAAAGTCACTTCC<br>CCCACCACCCGGACCACTAATGCGGACTTTGAGCTGGATAAAAGTCACAAAGTCATGATTAAAATTTTAAAGTTTTTTTAACTTAATCGA<br>TAGACATGCGCGTAAGATTAAAAAAATAATTTTTATAAATGTGGAATGACCACCCCTTCTATTCGAAAAACAACCCTAAAATTGACAAAAA<br>AAACCAAAATCTACAAATAAAGCCTAATCGATACGGTTTTTTGCATATAGGTAGCCCCTAAAATATTAATAAAAAATATTTTAGCAAAAAC<br>CACCCCTTTGTATTTGGGGGTGAGTTAAGGGTAAAAATGTAAAAATTAAAAAAAAAACAAAATTAACCAAATTTAAATATGTTGTGTATTT<br>TAGTGCGTAGATAAGGCTCTTATATAATTTTTTTAGATTTCACCCTTAGGGGGTGAGTTTTTAACGTATTTAAAAAACCACTAAATT	OQ551428
**TCsat25**	CTTTGATTTAAAAGTTTAGTTAAAAATTAGATGCTGACGCCTACTTTTGTGTAGGAGAACAATTTATTATACATTATCACGACGTATTTCTT<br>CTTAAGCAAAAATGGTGGATGCGCCGGACAAGCATCCAATATGCTGTACATTTTTGCCCTCCGAATAAATAAAATTTTTGCAAAGTGAAG<br>TAAAACTATATTATTTATTACGTAGCAAGTTTTTATGTAGATTGTTCGATATATTACACAAATTGCATAAAAAAAC	OQ551429
**TCsat26**	CTATTATTTAAAAAAACAAATAATTACACGTTACTTGTATAAAAGTACAAAGTTAAAGAATTTTTTGTTGTAACCATCACTGATTCTAAAAAT<br>AACTGAAAAGATTTAAAGTTAAAACGTTTTTATCTTCAAAGTAAATATAGCTGTTAAATTTTCTCTTTTCTTTTTTTTGCAGTGTTCTGCTTA<br>GTTTTCAAAAATTTTAATAACAAAATGCTTTAATAAAAATTACTAATATAACACATAATTAAAACTAAAACAAATATAGAACATGGTCTTGTA<br>CAATTTTGTCAACTCGGCGCCTCCACCATTTTTAATTTTAGATATCCAACCAATTTACTTTCCTTACTTTTACTGTAAATGA	OQ551430
**TCsat27**	GGTTCTTCACTTTGTGACTATTTTTCAAAGTTTTCTTGTCTGTTTTTGTCATATTTTGCGAATGAAATTTGGTTGTCCAAATGACATGAAAT<br>CTTGCATACTTACGTAATTTTCAAATCAGGAAGATCGAACCAACACCATTCGATGGAGTAGTACATATTTTTAATTCAAAGCTTCAAAAAA<br>CATTTGTTTATATGTTTTTGTGCACTTTTCACTTTATTGCGCCAAGAAAATTGTTGGTGGATTTACAGTAACGGTCCGCTTTTTTGTGTAAT<br>GCGCTCGCATTACTTTATATGTAAGTT	OQ551431
**TCsat28**	TAGTTTTAAATTGTATGGGAATTTTTTAGAAAATAGTTATATCTTCAAAAGAATGTAGCATTTGTAAAGACATCATTGCAAGTCTATAAAGT<br>CAAAAAGTACAAAAAATTTTGAACATTATTGTTTGGTAAAATTTTAAAATTAACAGACGGTTTTTTATTTTTTTTTATCAGGCAAAATTACTG<br>TAGGATACTGTAGGATTATAATTTATGGTAAATATACAGTTAAATACTTTAATGTGTTTATTATTAAGTACTTGTAAGAAAAACCTTTTATTT<br>TTGACTAAGCTTATGAAAAATTTATCACTTTTAAGTACAAAGAGTGTTTTATTTTAAAAAACTTACGTTTGTAGCGCAGCCAAGCATAAGA<br>AATTTGAGGGCCGCAAAATTTTCTACAATTTTTATTTTTTTTT	OQ551432
**TCsat29**	TTTTTTCTATCAAGGTTTATAGTCAAAAAACCTTAATTTCCCACTTTTCTTATGTAAAATTGTCTGAGAATTTCGAATTTGTTTTTAGTTTCT<br>ACCTGCGACTAATAGTTTTCGAGATATCGGGCAAAATTCAGTTCGTGAAGGTAAAAAACTTGAATGAGCGGTCTATTCATAATT	OQ551433
**TCsat30**	ACGGAAGAAGAAACAACTGCTAGTGGCACTGAAACTGGTGGAGGAGCTAATGTAGAAGAAACAAAAACTTCAACAATTGAAGAAGAAAA<br>G	OQ551434
**TCsat31**	CTTTTTTTTCTTCTTTTTAATTGGGGTTGGTGTTCTGGCTTCGAGTCCATTTCACCAGACGTATAGTTTTTACGCAAAGCATTGTTTGTTT<br>CAGAGTCAGAAATAATTAAAAGAAATATTTTTTAGCAAATCAAAAAAGTTCTT	OQ551435
**TCsat32**	CTTGGAGTTGCAAAATGTCGAAAGGGATTTCGAACCAGGATCACAAGAATGCTAAGAGGTGAAACGATGTCGACACCTCTTTGCGTTCA<br>TGTGATCCTAATGCAAATTTTAGTTTTAAATTATTAGAAAAAAAAATAATTTTTTAAGCTTCTGTATAATTGGTTGTCTATATAAGCTGTCAAT<br>TGTAAATTTTAGAAATGAAAACTCGTATCAGGATCACAGAAACGCAAGGAGGTCTTTAACTTTAAAGACCCTTTGACGTTCCTGTTATCC<br>TAATACCAATTTCGATTTAAAAATTTCGCAAATAAAAAAAATTATTTTTTCGG	OQ551436
**TCsat33**	TTTTGACTCCAAAACAGAACATTTATCATTTATTTTGTGATTTTTTAGTGTGTTTTTGGGTCTCGACCTAGAAAAAGATGAAGTTATCTTAT<br>CTTTTTCCAGGTCGAAGCCCAAAAACTAATTATTTATGTCATCTAATTTTAGGTAAAT	OQ551437
**TCsat34**	ATGAAAATTGGTTCACAGAATCATATTTAAAATAACATTTTACTCCAATGGACATTTTAAAAAATTGTCTTGGGACCAACCTAGATTTTCCT<br>TACCAGTTTTTGACAGTCAAAAGTTGAACTGCGACCGAAAATAGTAGTATTTCATGATTGTGTGGCAGTAAAAAAGGTCATTCTCTTGTC<br>TTTGTTTCCTCAAAAAAAAAT	OQ551438

**TCsat35**	AAAATATTAATTTTTTGTTAAACTTTTGAGACACCCTACCTACATAAACACTGTCTTTAAGCTTAAAAATCATCAATATACCTACCTAGAATTTTATTATTTTCATTTTATTTTATTATTTTATTACTTCTGCACAGACAGTGTTTAAAAAACAATCAGAAAAAAAAATAAAA	OQ551439
**TCsat36**	ATTTTTAATATGTTATAGAAGAAGGTCTTCTGAAGCTGTTTTATAAAAAAGAAGTTCATTGCTCGATGTTATCCAATCAAGACAGCGGAATTTTTAACTTGGTGTTTTATTTGAATTTTTCAAGAATGGTTATTTTTGGCAGACTCTAACAAGAAAACCAAAAATAATATCTTTCCAA	OQ551440
**TCsat37**	ATTCGAAGCGCGAATCACTTTAAGTTTAAATTTAAACAAATTCAAATGAAAACGAACTTGAATGCGCTTTCACGGCCGGTTTGATTTATTTATTAAATTTTAATTTTGAATTTAAATCAAATTTATTCAAGTACTCGAAATAATTTGATTGCAATTAAGGGCCGGTTTACAGTAAGCGATTTAAATTTTAATTCCGAATTAAAGAATAATTTGTTTTCGTTTTCAATTTA	OQ551441
**TCsat38**	TACCAAACATGAATTTTTTTGGCCAAGTTCCACTCATTATTTTTGAACGCAATTTTGCAAACTAATAGACATTAAACAAAGCTATAATCGTACAAAAGCTCGTTTTAAAAGACAGTCGAGTTCGGAAGATACGAATTTTTGAATTTAAGTTAGGCAAAATCAGGTTTTTGCTATCTGA	OQ551442
**TCsat39**	TAATAAGCTTTGAAAAAATAACACATTTGGCGTGAAACCAAATAGTTTTTTTAACTTGTTATTTCAATTAAGTGCCAGAATATTGATAATTGTTTATAACAAACGTCAATTATTAATCAAACCAAAAATTATGGTAATGTGACAAAGTTTACGTAAGTGTGCAAAATTTCAGTTAGTTCGGACAACAA	OQ551443
**TCsat40**	CTCTTTTTATATAAAAAGCAATTTCTAAAACTTCTAATAAAAATGAAATCAGCAGTCGCACTTTACAGACTATCTTAAAATTGTACAATGACATAAGAGTACTGAAGAGTACTACTTTACGGACGATTTCAGAAAAAAACCTTTTAGCATCAGCAGTATTAACGTAGTAAATCAATCAGTATTTATTATAAAGAATAGTGAAAAAAATCGGAAAAAATCAATTTTGAGAAAAAGTTAAAATATATTTCAAATATTGTAAAAAAGTGATGAGGTTGT	OQ551444
**TCsat41**	AAGGACCCTTGAATAGTTTGCGAATAATTTTACTTTTTTTACAACAAATTCTTTATCTTAGTACCTGAACCTCTTACAGCTCAAATAAATTGGTTTTCAAGAGAAAAAAAAAATATTTGTGCG	OQ551445
**TCsat42**	GAATTACGAATTTTTTATTTTCTGATGTTGCTAATTTTTTTCTCGACTGTCGAAAGACAATTCGTTAAATTCTTATAACTGTTCTAGTGCACATTTTGTTTCGAATTATTCTGAGTTGGTAGACCAATATTTGAGGCACAAATAGCCAAAAAGGCGTTAAAAAATATTTGCTAGTTTCA	OQ551446
**TCsat43**	GGGGTGTCTACCAAAGTGATGACACCTGGTATTTTAACTACTTTCCCAAACTTTGACTGATATCTTAAAAATTAACAGCAATCTACACAGCCTTGAAAAATCCTTAAAAAAATATTGCTAAACCTTTCGCTAAAACCCTGAATATGTCTTTTTATACTTTTTTAAATAAAAAAGTTAG	OQ551447
**TCsat44**	TATAATATCACTTCCGTTTGGCGATTTTTCGGATTTTTTTGGGGCTCGGTTTAAAAAATTCGCTTAACAATGACGTTTTTGGATGGAAAATGTTTTACGAAAGCATATTTCATTAAAAAAAGTGACATTTATTTCTGAAAGGGGGTTCATTTTCTGAACGAGAATTTATAAACGTTCAGGCTCAGTCGTGGTCGGGTCCTAAGAATTTTCTTACTTTTGCTAAAGTAAAATAAGTTTGCCATTTTTTGTTAGTATTTGCCGAAATTTCGGAAAATAATTCAGAACAAAAACGTGCATAAACGGCTGAAAAA	OQ551448
**TCsat45**	CTTTATTTAAAAACTTTTGTCTATCACACTATTTGGATTTTTTAATCCAATTTTTTTAAATTTCTGTTTATGGCCATTATTCATTATTCTAACGAAAAATAACACTCAGACCTGAAACTATAAAGCAATAAAAAGCTAATTTTTGACTGAACGGAAAAATGTATCTCGGAAATGTTTTGTATTTTTTAGGAAAATTTCTCCAACAAATAAATATTGTAGTGCATTTAATTGATTTGGTTCGGTTTCACTAAAAACTGCCA	OQ551449
**TCsat46**	TCAAACACTATGAAAAAGCCTACAATAAGTAGAATGAACTCTAGAATTCTCTGAAAAAAACGTTTTGCTTTTATTTTTTGGGTTTTAAGGTAGATAATTATCACTGTATTCGTAATTTTATAAAAAAAATATAAAAAAAAGC	OQ551450
**TCsat47**	TCTACTTTTGTATTTTGAGAAATAACGTTTTGAATATTTTATTCACTTTATTCGTATTAATAAAGAAATTTCTAAATAAGTTGTTACATCTGTTCTTTATTTTTTATCAATCATTTTAATAATCTGATTAATAATAATGGATACACACCATAAAGCGACATATTTTTATCAAACCTTTTTCCTCATCTGCTCTTGCTTGCGTCTCTTAATTAGAAGATTAGAAAAATAAGTGAAAGGTAAAAATCAGAAGATATAGAAAAATTTAGGAAACAAAGTGGAGTT	OQ551451
**TCsat48**	GAAAAAAAATTACAGAGAAATTTTCGAAAGTACAGTGGAACCTTGATAATCCAAGCTAAGAAAAGAAAAGCTTAATCTTGTTTTACTCAAATGACACTATTTTTATAGAAAAAATGCTTAAAAGCAAACGATTTTTGCGAGATTTATTTATTTACT	OQ551452
**TCsat49**	AAAAAAATTCTTCTGATAAAGTTAGATACGAATAAAAAAACTAAGTTATTGGTTTTAATTAATTAAATAATTAATTGACGTTGGAAGAGTTATTTTACATTTATAATCGATATTTTCTTAAGACAAGTAAGCTAAGGAGTCAAGGATTTTATTGCTGAAGGTAATATGAGCAGTTTAAAAAATATTAACAAAAAAACAGTTTTTGAGTGAACTTTACACTCGAATTTTGAACAAATAGTAAAACAAAAATTTATTTTTTTTATGTTAAATTAACCAAAAAATCCAAATCTGCAAAAATTATTTACCTACGACGAGAATTTTCGGAAGAGAAATGCAAATCAACAAAAACAAACT	OQ551453

**TCsat50**	CTTAATTATTTTTTGACAAACTTACTATTTAAGAAACATAAATTAATCACAATCTGTTATAACTTAAAAAAAATTAAAAATGTCCTTAGGTAA<br>CAAATTATTTACTTTGGCCGTAATTAAAAAATCGCCCAGTATTTCAAAACGATTTTCTAGAAAATAAACAATCAC	OQ551454
**TCsat51**	GTTAACTTAAACCCAATTTAGAGTCGAATTTGACTAAAAAAAATTTTTAATTCAATAAATAAAAACTTGGTAAAATTGACTTCGCTTATTTAT<br>TCAGTGATATCTTCGTAATTACTAAGTTTATGATCAAAATAATAACATATTTGGAAAATATGGACAAATTTACGTAAGAAAAGCTTAATTTT<br>AACCCAATTTTTAGTTTTATTTTATTAATACAAATTTTCGAATTTATTATTTAAAAACTTGGCAAAACCAGCTTCACTTATTTATCAACCGAT<br>ATCTTAAAAATTACTCAATTTATGGCTAAACTAATTACAGATTTAAAAAATATGATCAATTTTACGTTAGGAAAA	OQ551455
**TCsat52**	CACTTTGTAGAGAATTTTATTGTCTTTAATTTTGTTCCTTACTTATTTGGTCGTAACACGTGTCGTTTTCGAGTTATAGGTGAAAAAATCG<br>GAAACGGCACTTTTTTGAGCTCACTTCTAGAATCAAAGACTTTGACAGCGGATATCTTCAAAACTATTTGCGACAAAAAAAAAGTGAAAA<br>CAC	OQ551456
**TCsat53**	TGTTTTCCTTTTTTCAGTTATATTTTATTCTTTTTTAGTTTCTCGGGCATTTATTGTTTTATTTTCTCTTAATCTCGGTTATTTTACTTTGCAG<br>TTTTTAAGTTAAACTTCTCATGGTTTAGACTATTTTTTTCTCATTTTCAGCATTTTTA	OQ551457
**TCsat54**	AAGGTTTTGCTACAAATATTTGTATTAAATAATGAGAAATAGGTGAAAATCAAGCGAATAAAAATGAACTTACAATTAACAGACAGTTTTT<br>CGTAATTTTTCAAACATTTTGACGTAAGTTTTTGAAAAAAATGACTGGTTTTCAAAGTAGTTTTAACTAATTTTCGTACAATTTTTTTATTAT<br>TCCTTTGGAAATTTTGCAAAACAATCTTGTATTGAGTTATTGTAGAAAAAAATAACGACAGTTTTTTGTATTTTTTTTTAAATTTTGACTTTTA<br>TTTTACCAAAAAATATTGTTTCTTATTTAAGATTGCGCAATTCTG	OQ551458
**TCsat55**	AAAATTAATCCCTTTATTCTAGAAACTAGAATTTCAAAATTCTGGAATTCTAAATGACACTAGGTATAGCAGGCTATTTAGAATTTTGCTAT<br>TCTGATATTTTGCTTTGGAAGTTTAGAATTCTATACTCGAAACATTCTACGAAAAAATTTAAGATTCTAAAATTCAAAATATGTACCCAAAG<br>TTTTCGAAACTTTAGACTTGAACATGTGAACTTTGAAACTTCTAAACTTCTAAATAACTAAAACTTGAAGATCTAAAGTTTCAAAGTTGTTC<br>TAAGTTTCAAGATTAGGTACAATTTCTAAGATTCT	OQ551459
**TCsat56**	AGCTATACAAGTACGAAATATTTTGCAATAATTACAAGATCTTGCCAAAAATTATGCCAAAAATTGTTTTTAGTCTATCTCTAGCATGCTAA<br>ATAACTAGCTTAATGAGTGGGTGTTAAATACCTAGGTAAGTATAGCGTATTATTATTTTTTCTTAGCAAATCGTATTTGAAAATACGTTGC<br>CACTTAACATTTAAATTATTTTACCAAGCATTTACAATAATTTACTCAGAAGATGTCTATTATTTACCTTTTTTACTTGTTTGATCCAATCGC<br>TAAAAATGGTGGAGGCGCCGGGAAATAATT	OQ551460
**TCsat57**	CTTTTTTTACAAAAAATATTTTTAAGTATACTGAGTGCTTTTCAATTTTGCTACAATACAAAATTATGTTTTTTTTAAATAGAAAACTATATAG<br>GTATTTTTGGATTTCGATCGATTTTTATTAATTTTTGAAACAATTTGTTTTTTTTTTCACAAAAACAAGCTTTTTTGAAATTTATTATATTAAA<br>ATTAAGAATCCTAAAACAGGAGGA	OQ551461
