# Supplementary material for: The Low-Copy-Number Satellite DNAs of the Model Beetle Tribolium castaneum
Source: Genes (Basel). 2023 Apr 28;14(5):999. doi: 10.3390/genes14050999 (PMC10218199; doi:10.3390/genes14050999)
Supplement: Supplementary file 1 [file genes-14-00999-s001.zip › Supplementary materials/Table S3.pdf]

Table S3. Main characteristics of the 46 *T. castaneum* satDNAs identified in this work. The data on monomers' length, AT content, nucleotide divergence, and tandem/scattered organization have been based on the repeats annotated in the current reference genome of *T. castaneum* [a]. The names of satellite DNAs whose repeat units were found only in tandem organization are shaded in gray.

SatDNA	Genomic abundance[b] (%)	Monomer consensus length (bp)	Mean monomer length (bp)±SD (min-max)	AT content (%)	Nucleotide divergence (%)	Expected No. of monomers[c]	No. of annotated monomers[d]	No. of repeats in arrays of ≥5 tandem copies / No. of arrays with ≥5 tandem copies	No. of scattered repeats	No. of tandem repeats in the longest array	Partial similarity to Repbase entries	Annotation according to *Wang et al.*[e]
**TCsat12**	0.0461	154	152.6±3.2 (142-157)	68.7	10.5	611	82	78 / 4	4	39	-	-
**TCsat13**	0.0184	266	266.8±4.4 (263-288)	69.6	12.4	141	50	50 / 4	-	21	-	-
**TCsat14**	0.0154	309	309.9±1.1 (307-312)	68.2	1.3	102	37	35 / 2	2	24	HAT-14_Mad	-
**TCsat15**	1.6857	1106	1100.9±15.4 (1014-1163)	68.1	19.5	3109	330	6 / 1	324	6	Academ-3_LMi Transib-N1_CQ Sola1-2_LMi	R=0|TRF=0.08 | NSEG=0.370| HighB
**TCsat16**	0.0059	330	329.6±1.9 (319-330)	64.5	6.3	36	34	34 / 1	-	34	DNAX-1_TCa Dada-tV_PeFlu	-
**TCsat17**	0.0077	120	120.0±0.4 (117-120)	60.3	11.0	131	70	68 / 2	2	36	-	-
**TCsat18**	0.0892	73	71.7±4.3 (61-83)	64.0	25.4	2493	969	424 / 23	545	84	-	-
**TCsat19**	0.0174	618	606.0±22.7 (553-639)	68.6	13.7	57	43	31 / 4	12	11	MuDR-7_Cas	R=121|TRF=0. 000 |NSEG=0.328| Mid
**TCsat20**	0.0045	144	144.5±1.7 (143-150)	68.3	2.3	64	24	24 / 1	-	24	-	-
**TCsat21**	0.0041	102	102.4±1.0 (101-105)	70.8	4.2	82	30	30 / 1	-	30	HipGal-6.520	-
**TCsat22**	0.0040	281	280.9±0.3 (280-281)	69.9	1.5	29	20	20 / 1	-	20	-	-
**TCsat23**	0.0078	164	162.9±3.5 (135-165)	70.1	7.4	97	68	58 / 8	10	9	Rehavkus-1_TC	R=3600|TRF= 0.000|NSEG= 0.382|Mid
**TCsat24**	0.0898	721	718.1±19.9 (653-762)	67.1	10.9	254	23	6 / 1	17	6	CerCot-5.769 EnSpm-N2_DaCa	R=242|TRF=0. 000|NSEG=0. 342|Mid
**TCsat25**	0.0027	258	258.2±1.5 (256-261)	70.7	5.9	21	25	25 / 1	-	25	-	-
**TCsat26**	0.0030	361	363.0±1.1 (361-365)	77.2	6.8	17	20	20 / 2	-	10	Transib-3_DTa Gypsy-54_MT-I	R=4675|TRF= 0.000|NSEG= 0.433|Low
**TCsat27**	0.0040	302	298.7±11.0 (248-304)	67.7	9.5	27	48	48 / 2	-	24	Gypsy-22_DMel-I	-
**TCsat28**	0.0036	412	410.7±4.7 (400-414)	76.4	5.2	18	7	7 / 1	-	7	MuDR-18_Cas EnSpm-14_RIr	-
**TCsat29**	0.0028	177	176.1±0.6 (174-177)	70.8	11.8	32	44	42 / 2	2	21	-	-

**TCsat30**	0.0019	90	90.4±1.3 (90-96)	59.2	19.7	43	29	25 / 2	4	17	-	-
**TCsat31**	0.0018	144	144.6±2.1 (140-148)	70.4	4.5	26	25	25 / 1	-	25	-	-
**TCsat32**	0.0033	325	308.8±35.1 (230-326)	69.5	10.4	21	18	18 / 1	-	18	-	R=2222|TRF= 0.000|NSEG= 0.308|Mid
**TCsat33**	0.0017	150	149.7±0.7 (148-150)	72.0	8.1	23	6	6 / 1	-	6	DNA/Mariner	-
**TCsat34**	0.0020	203	203.6±1.1 (202-206)	69.7	3.0	20	9	9 / 1	-	9	-	-
**TCsat35**	0.0018	172	172.4±0.8 (172-175)	79.2	3.2	21	13	13 / 1	-	13	-	-
**TCsat36**	0.0020	178	178.2±1.3 (175-181)	72.6	11.1	23	13	10 / 1	3	10	Helitron-N2_HMM	-
**TCsat37**	0.0470	230	258.9±45.4 (195-347)	71.6	33.9	417	235	115 / 8	120	51	Mariner-32_RPr	-
**TCsat38**	0.0070	178	177.8±2.4 (170-181)	71.0	26.1	80	88	46 / 2	42	23	-	R=1576|TRF= 0.000|NSEG= 0.081|Mid R=3912|TRF= 0.000|NSEG= 0.103|Low
**TCsat39**	0.0018	188	191.7±3.8 (188-197)	73.0	7.4	20	26	26 / 1	-	26	-	-
**TCsat40**	0.0020	276	275.8±1.6 (272-278)	74.5	5.0	15	10	10 / 1	-	10	-	R=4888|TRF= 0.000|NSEG= 0.000|Low
**TCsat41**	0.0016	122	122.1±1.1 (120-123)	71.4	7.1	27	20	20 / 1	-	20	-	R=4460|TRF= 0.000|NSEG= 0.314|Low
**TCsat42**	0.0016	179	178.2±3.6 (162-180)	70.7	11.2	18	22	19 / 1	3	19	Gypsy-7_TCa-I	-
**TCsat43**	0.0016	178	177.9±0.3 (177-178)	69.4	3.3	18	18	18 / 1	-	18	ARNOLDY2	-
**TCsat44**	0.0021	311	307.7±5.2 (296-312)	68.4	3.2	14	16	16 / 1	-	16	-	-
**TCsat45**	0.0017	260	260.0±0.6 (259-261)	73.5	4.3	13	6	6 / 1	-	6	COPIA3-I_MT	-
**TCsat46**	0.0017	142	141.9±0.9 (140-143)	76.6	4.4	24	15	15 /1	-	15	-	-
**TCsat47**	0.0019	282	283.8±5.4 (281-297)	76.0	6.8	14	13	11 / 1	2	11	hAT-N20_ALy	-
**TCsat48**	0.0017	156	149.9±10.9 (130-159)	72.4	15.8	22	7	7 / 1	-	7	Polinton-2_HM	-
**TCsat49**	0.0017	354	354±3.7 (350-361)	76.4	6.8	10	5	5 / 1	-	5	Gypsy-6B_AlBr-I hAT-26_DTa	-
**TCsat50**	0.0024	168	168.3±0.7 (167-170)	77.4	14.0	29	19	19 / 2	-	12	Gypsy-45_PAb-I	-
**TCsat51**	0.0043	353	352.4±4.2 (340-354)	76.9	13.2	25	10	6 / 1	4	6	Gypsy-671_AA-IMuDRx-3_TV Helitron-N60B_CGi	-
**TCsat52**	0.0013	184	183.8±1.4 (181-187)	66.8	19.8	14	34	34 / 4	-	10	Academ-10_LMi	-
**TCsat53**	0.0017	152	151.9±2.3 (149-157)	74.9	11.8	23	11	11 / 1	-	11	MuDR-2_GAr	-

**TCsat54**	0.0022	322	321.3±1.6 (318-323)	77.6	8.0	14	9	6 / 1	3	6	HARB-N1_TC Gypsy-122_NS-LTR Gypsy-27_DWil-I	-
**TCsat55**	0.0023	311	311.0±0.0 (311)	73.3	3.9	15	5	5 / 1	-	5	DNA-1_CQ	-
**TCsat56**	0.0024	306	306.2±0.4 (306-307)	70.8	5.3	16	5	5 / 1	-	5	Harbinger-21_OS	-
**TCsat57**	0.0013	212	210.2±2.0 (208-214)	80.1	4.1	13	5	5 / 1	-	5	-	-

[a] Herndon et al (2020) BMC Genomics 21:47

[b] An average value of genomic abundance based on TAREAN analyzes shown in Table S1.

[c] Expected number of monomers in the genome was calculated according to the estimated genome size of *T. castaneum* of 204 Mb (Brown et al (1990) Insect Biochem 20:185-193) and the TAREAN estimates of satDNA genome abundance.

[d] Monomeric repeats annotated in the genome assembly Tcas5.2, as well as in unplaced scaffolds (GenBank accessions DS497665-DS497969) and unplaced singletons (GenBank accessions GG694051-GG695898).

[e] Wang et al (2008) Genome Biol. 9(3):R61.
