# Supplementary material for: The Low-Copy-Number Satellite DNAs of the Model Beetle Tribolium castaneum
Source: Genes (Basel). 2023 Apr 28;14(5):999. doi: 10.3390/genes14050999 (PMC10218199; doi:10.3390/genes14050999)
Supplement: Supplementary file 1 [file genes-14-00999-s001.zip › Supplementary materials/Table S6.pdf]

Table S6. Primers and PCR amplification conditions specific for satDNA TCsat12, TCsat13 and TCsat14.

	**TCsat12**	**TCsat13**	**TCsat14**
**Forward primer**	AGAAAGTGGCACATTGTCGAT	ATTCAAATAAAGGGGGTAGTG	TTACAAACGGAACAGATGA
**Reverse primer**	AAAAATCGAGCCCTGGGAAG	TTGGGGTAGGAAATGAGAA	TACAGGCTAAAATACACTTC
**Annealing temperature**	43°C	46°C	53°C
**The length of PCR amplified monomer**	99 bp	143 bp	107 bp
**The length of PCR amplified dimer**	253 bp	409 bp	416 bp
**The length of PCR amplified trimer**	407 bp	675 bp	725 bp
