# Supplementary material for: The Low-Copy-Number Satellite DNAs of the Model Beetle Tribolium castaneum
Source: Genes (Basel). 2023 Apr 28;14(5):999. doi: 10.3390/genes14050999 (PMC10218199; doi:10.3390/genes14050999)
Supplement: Supplementary file 1 [file genes-14-00999-s001.zip › Supplementary materials/Table S7.pdf]

Table S7. Origin and number of TCsat12, TCsat13, and TCsat14 monomers used in phylogenetic analyses.

	**TCsat12**	**TCsat13**	**TCsat14**
Cloned PCR-dimers (containing one full-size monomer)	*13*	*24*	*12*
Cloned PCR-trimers (containing two full-size monomers)	*9*	*5*	*17*
Extracted PCR monomers	31	34	46
Monomers from assembled contigs	4	-	36
Monomers from unplaced scaffolds	7	19	1
Monomers from unplaced singletons	71	31	-
**Total number of analyzed monomers**	**113**	**84**	**83**
